# Global 5mC and 5hmC DNA Levels in Human Sperm Subpopulations with Differentially Protaminated Chromatin in Normo- and Oligoasthenozoospermic Males

**DOI:** 10.3390/ijms23094516

**Published:** 2022-04-19

**Authors:** Marta Olszewska, Oliwia Kordyl, Marzena Kamieniczna, Monika Fraczek, Piotr Jędrzejczak, Maciej Kurpisz

**Affiliations:** 1 Institute of Human Genetics, Polish Academy of Sciences, Strzeszynska 32, 60-479 Poznan, Poland; marzena.kamieniczna@igcz.poznan.pl (M.K.); monika.fraczek@igcz.poznan.pl (M.F.); 2 Faculty of Biology, Adam Mickiewicz University in Poznan, 61-614 Poznan, Poland; oliwiakordyl@gmail.com; 3 Division of Infertility and Reproductive Endocrinology, Department of Gynecology, Obstetrics and Gynecological Oncology, Poznan University of Medical Sciences, 60-535 Poznan, Poland; piotrjedrzejczak@gmail.com

**Keywords:** sperm methylation, sperm hydroxymethylation, oligozoospermia, male infertility, sperm chromatin, protamines, semen parameters, 5mC, 5hmC

## Abstract

Epigenetic modifications play a special role in the male infertility aetiology. Published data indicate the link between sperm quality and sperm chromatin protamination. This study aimed to determine the relationship between methylation (5mC) and hydroxymethylation (5hmC) in sperm DNA, with respect to sperm chromatin protamination in three subpopulations of fertile normozoospermic controls and infertile patients with oligo-/oligoasthenozoospermia. For the first time, a sequential staining protocol was applied, which allowed researchers to analyse 5mC/5hmC levels by immunofluorescence staining, with a previously determined chromatin protamination status (aniline blue staining), using the same spermatozoa. TUNEL assay determined the sperm DNA fragmentation level. The 5mC/5hmC levels were diversified with respect to chromatin protamination status in both studied groups of males, with the highest values observed in protaminated spermatozoa. The linkage between chromatin protamination and 5mC/5hmC levels in control males disappeared in patients with deteriorated semen parameters. A relationship between 5mC/5hmC and sperm motility/morphology was identified in the patient group. Measuring the 5mC/5hmC status of sperm DNA according to sperm chromatin integrity provides evidence of correct spermatogenesis, and its disruption may represent a prognostic marker for reproductive failure.

## 1. Introduction

Epigenetic modifications are well-known reversible variations of the genome that determine the transcriptional status of the cell. Epimodifications are mostly prone to environmental factors and are heritable [1,2,3]. The primary epigenetic mechanisms involved in the regulation of the genome are DNA modifications (i.e., methylation), posttranslational modifications of histones (i.e., methylation, acetylation, ubiquitination, phosphorylation, etc.), and the activity of non-coding RNAs (nc-RNAs) [1,3,4,5,6,7]. The best-known epigenetic mechanism is the methylation and demethylation of the 5’ cytosine. Primarily, 5-methylocytosine (5mC) can be observed in CpG islands, where these moieties remain for the majority methylated (60–80%) [7]. DNA methylation remains stable in particular tissues, each type of which has its own pattern, but can be influenced by disease, pathological events, or age [7,8,9,10]. Cytosine methylation determines the genome-wide methylation pattern of DNA and the modification of histones, resulting in changes in chromatin configuration, playing a crucial role in gametic imprinting, gene silencing, chromosome X inactivation and protein conformational changes [1,3,4,6,7,11]. In the non-CpG context, DNA methylation has been observed in oocytes, embryonic stem cells and some types of brain cells. In contrast to methylation, hydroxymethylation (5hmC) is present at lower levels in particular tissues (0.1–0.8%), exhibiting increased values in tissues with high transcriptional activity (i.e., neurons) [7,12,13,14]. This epimark, a result of enzymatic oxidation of 5mC to 5-hydroxymethylocytosine (5hmC), is mostly observed in enhancers, promoter regions of genes, and other transcriptional regulatory elements [7,15]. In spermatozoa, which are transcriptionally inactive, the level of hydroxymethylation is approximately four times lower than in somatic cell types [16]. It has been suggested that 5hmC, together with Tet family enzymes (Ten-Eleven Translocation Proteins; Tet1, Tet2, Tet3), may supervise gene expression through regulation of methylation [12,14,16,17,18,19]. Thus, both 5mC and 5hmC seem to play important roles in proper genome function.

It is well established that parental genomes play distinct genetic roles after fertilization. Functional sex-determined differences result from gametic imprinting established during gametogenesis. Thus, each parental genome has a different methylation pattern that imposes key epigenetic mechanisms in the proper development of an organism, beginning during gametogenesis [3,6,20,21,22]. The paternal genome is responsible for early placental development, while the maternal genome is responsible for embryonic development [3,20,21,22]. It is also known that in newly formed embryos, certain developmental disturbances may occur due to a lack of activation of genes crucial for normal development, which can be linked to disturbances in proper methylation and demethylation rounds in gametogenic cells, and to disrupted methylation/acetylation of sperm histones [3,21,23,24,25,26,27].

Chromatin of spermatozoa is uniquely organized and condensed (4-6 times stronger), which results from overlapping toroidal structures built from DNA and protamines (enriched in arginine and cysteine residues, and thus positively charged), containing only a residual quantity of histones [28,29,30]. The condensation of the sperm chromatin generates a genetically inactive state which is crucial for the fertilization process when transportation of paternal genome occurs [28,29,30]. In properly protaminated human sperm chromatin, only approximately 10–15% of gonadal histone variants remain associated and are highly acetylated [1,31,32,33]. Importantly, this epigenetic mark can be transmitted from sperm to oocyte and may be involved in the regulation of gene expression in early embryos [1,3,34]. Additionally, the remaining histones are in contact with the nuclear matrix. These regions may contain gene promoters that are among the first to be transcribed after fertilization, including microRNA-coding sequences and imprinted genes [1,2,22]. The remaining histones are also located on the unmethylated DNA within genes associated with spermatogenesis [33,35,36]. Importantly, the presence of histones forces nucleosomal packaging of chromatin, that takes place between DNA-toroid complexes. Thus, regions containing histones are potentially more prone to chromatin disruptions caused by free radical attack, mutagens or nucleases [32]. It has been documented that disrupted expression in a proportion of protamines P1:P2, as well as in the ratio of protamines to the remaining histones, were implicated in male infertility [37,38,39], revealed in reduction of sperm quality or induction of sperm DNA damage [37,38], leading to breakdown in embryonic development [25,27,40]. It was also documented that apoptosis of sperm cells was linked to chromatin deprotamination and sperm DNA fragmentation, which was then associated with spermatogenetic disorders, manifested as hypermethylation of the genome [41,42], which may be an early response to oxidative stress mediated by an increase in the activity of DNA methyltransferases (Dnmt) [43]. Additionally, oxidative damage of sperm DNA may be at least in part responsible for changes in global sperm DNA methylation [41]. The hypothesis that spermatozoa with hypomethylated DNA may be more prone to DNA damage is well known [41]. Taken together, these data support the hypothesis that in spermatozoa with decreased chromatin integrity, global sperm DNA demethylation may be increased.

Investigating immature spermatozoa collected for fertilization in assisted reproductive technologies (ART) revealed that it is possible that inadequately established methylation patterns and improper chromatin integrity can increase the risk of reproductive failure or future offspring health status [1,3,41,44,45,46,47,48,49,50,51,52,53,54,55,56,57]. Hypomethylation may also alter the process of cellular differentiation, so embryonic genome expression may reveal disturbed synchronization in its development [3,22,58,59]. Consequently, the unique epigenetic marks in spermatozoa may be crucial in facilitating proper mature gamete function, and are responsible for poising specific gene activation in the early embryo [1,3,27,46,48]. In this light, basic knowledge concerning the mechanisms and meaning of gametic epigenome disturbances seems to be important, due to the relatively high frequency of ART births today (approximately 1–3% of all live births) [48,60,61].

It is known that male infertility may be linked with aberrant DNA methylation in human spermatozoa. This was confirmed by changes in DNA methylation both in global sperm DNA and in particular genes (imprinted or nonimprinted). Changes in the methylation pattern were also documented for males with disturbed protamines P1/P2 ratio with respect to sperm apoptosis, in IVF patients, in response to male ageing, in chromosomal aberration carriers, and in patients with decreased semen quality [22,42,44,45,48,62,63,64,65,66,67,68,69,70,71,72]. In cases of oligozoospermia (decreased sperm count in ejaculate), only 5% of oligozoospermics are able to fertilize, and genetic causes are responsible for 2.5–10% of observed cases (i.e., microdeletions in chromosome Y) [73]. Epigenetic evaluation of oligozoospermia revealed that abnormal methylation patterns or imprinting errors in some patients from this group may lead to decreased fertilization efficiency and increased abortion rates [40,48,59,74,75,76,77,78].

The aim of this study was to discover whether there is a correlation between the defined status of sperm chromatin deprotamination and global 5mC and 5hmC levels of sperm DNA. For the first time, a sequential staining algorithm of the same human spermatozoa was applied, allowing researchers to determine three sperm subpopulations according to their chromatin protamination, with subsequent estimation of 5mC and 5hmC. Additionally, the sperm DNA fragmentation level (TUNEL assay) was examined to support the chromatin integrity data. Comparison of two groups of males with different semen parameters allowed observation of possible correlations with sperm abnormalities. The study is supported by a review of literature data published so far concerning methylation and hydroxymethylation in spermatozoa.

## 2. Results

### 2.1. Semen Parameters

**Figure 1:**
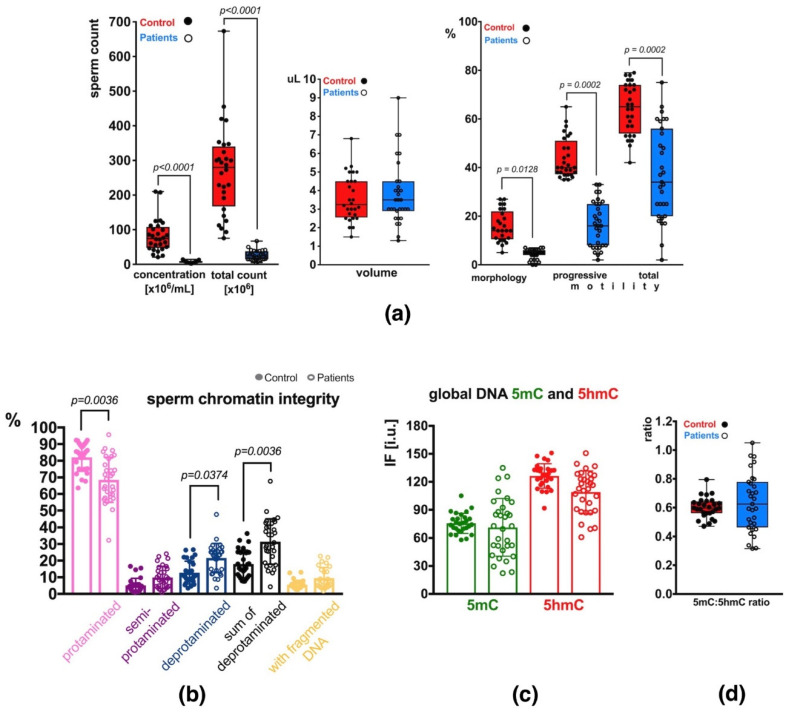
Sperm parameters observed in the control (C) and patient (P) groups. (**a**) Semen parameters; (**b**) Sperm chromatin integrity parameters (protamination—measured by aniline blue staining, and DNA fragmentation—measured by TUNEL assay); (**c**) Mean global DNA methylation (5mC) and hydroxymethylation (5hmC) status measured for the total, unfractionated sperm population; (**d**) 5mC:5hmC ratio in the unfractionated sperm population. Bars represent: (**a**)—upper and lower quartiles, whiskers: mean to max values and all measured points; (**b**–**d**)—mean values ± SD and all measured points. Statistical significance was considered at *p* < 0.05.

Semen analysis of the group of patients (P) revealed decreased basic sperm parameters in all (31) individuals compared to the control group (C) with normozoospermia (Supplementary Table S1). In total, 2 patients exhibited decreased sperm count only (oligozoospermia, O), 22 had decreased sperm count and motility (oligoasthenozoospermia, OA), and 7 had decreased sperm count, motility and morphology (oligoasthenoteratozoospermia, OAT) (Supplementary Table S1). Statistical differences in values obtained in each studied category are presented in Figure 1a. Statistical significance between both groups of males (C vs. P) was observed for sperm concentration per mL (*p* < 0.0001), total sperm count (*p* < 0.0001), sperm morphology (*p* = 0.0128), and sperm motility (progressive: *p* = 0.0002, total: *p* = 0.0002) (Figure 1a). The ejaculated sample volumes were similar in both groups. The mean P sperm parameters were significantly lower than the values in the C group and showed wider ranges of values (increased heterogeneity) (Supplementary Table S1; Figure 1a).

### 2.2. Sperm Chromatin Integrity

The results of aniline blue (AB) staining showed that the mean frequency of spermatozoa with properly protaminated chromatin obtained for the analysed group of patients (P) was 68.56 ± 13.59% (range: 32.28–95.74%), and was significantly lower (*p* = 0.0036) than the mean control (C) value of 82.02 ± 8.31% (range: 63.60–92.37%) (Table 1 and Table S2, Figure 1b). Results of the TUNEL assay (Table 1 and Table S2, Figure 1b) showed that the mean frequency of sperm with DNA fragmentation in the patient group (P) was 9.55 ± 6.29% (range: 1.81–22.70%) and was 1.73-fold higher but not statistically significant when compared to the mean C value of 5.52 ± 2.62% (range: 2.50–13.06%).

### 2.3. Global Methylation (5mC) and Hydroxymethylation (5hmC) of Sperm DNA

#### 2.3.1. Unfractionated Total Sperm Population

To quantify global m5C and 5hmC levels in sperm DNA, the immunofluorescence (IF) technique was applied. In the total unfractionated control sperm population, the mean control result for 5mC was 75.61 ± 10.69 i.u. (range: 57.97–105.06 i.u.; Table 1 and Table S2), and was statistically similar to the mean obtained for the patient group: 71.32 ± 30.82 i.u. (range: 22.18–134.98 i.u.; Table 1 and Table S2, Figure 1c). In the case of 5hmC, the mean control value of 126.33 ± 13.17 i.u. (range: 91.71–150.98 i.u.) was also statistically similar to the mean P result of 109.03 ± 22.74 i.u. (range: 60.89–150.80 i.u.; Table 1 and Table S2, Figure 1c), even when the difference between values was 15.55%. Additionally, the 5mC/5hmC ratio was calculated, and no statistically significant difference was noted between the two groups (mean C: 0.60 ± 0.07, range: 0.47–0.79; mean P: 0.64 ± 0.20, range: 0.32–1.05). A wider range of values was observed in the P group (Table 1 and Table S2, Figure 1d).

#### 2.3.2. Sperm Populations According to Chromatin Protamination

**Figure 2:**
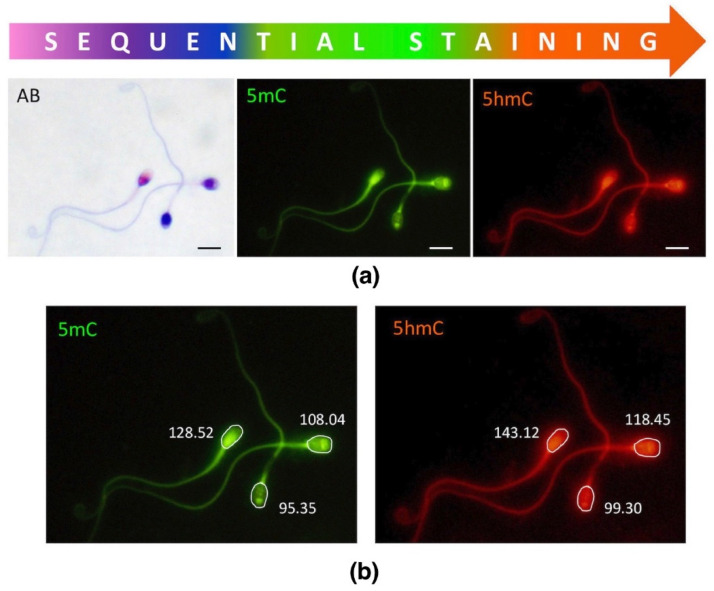
Sequential staining of three sperm subpopulations, including sperm chromatin protamination status and global levels of sperm DNA methylation (5mC) and hydroxymethylation (5hmC). (**a**) An example of the staining results of three different sperm subpopulations; AB—aniline blue staining: pink (unstained) with proper chromatin protamination, purple (partially stained) with disturbed protamination, and navy blue (stained) with deprotaminated chromatin; 5mC—5-methylocytosine (primary antibody used: anti-5mC, clone 33D3, MABE146, Merck, Temecula, CA, USA; 1:200; secondary conjugated with FITC (F2012, Sigma-Aldrich, St. Louis, MO, USA; 1:400); 5hmc—5-hydroxymethylocytosine (primary antibody used: anti-5hmC, ab106918, Abcam, 1:1000; secondary conjugated with AF594 (ab150160, Abcam, Cambridge, UK; 1:800)). Microscope: Olympus BX40, CellSense Dimensions; Leica DM5500, CytoVision; magnification 1000×, oil immersion objective 100×; filters: DAPI/Triple/FITC/SpO; bar represents 6 μm. (**b**) An example of single measurement values generated by the software (CellSense Dimensions, Olympus).

In this study, we applied sequential staining algorithm to the same spermatozoa (cell by cell, in situ on a microscopic slide as indicated in Figure 2), which allowed us to acquire and collate all the results from the same individual sperm cell. First, spermatozoa were stained with AB for chromatin protamination evaluation, followed by documentation of their position on microscopic slides. Then, immunofluorescence staining (IF) was applied onto the same slide, and epimark analysis was performed according to the documented positions of each spermatozoa from AB analysis.

**Figure 3:**
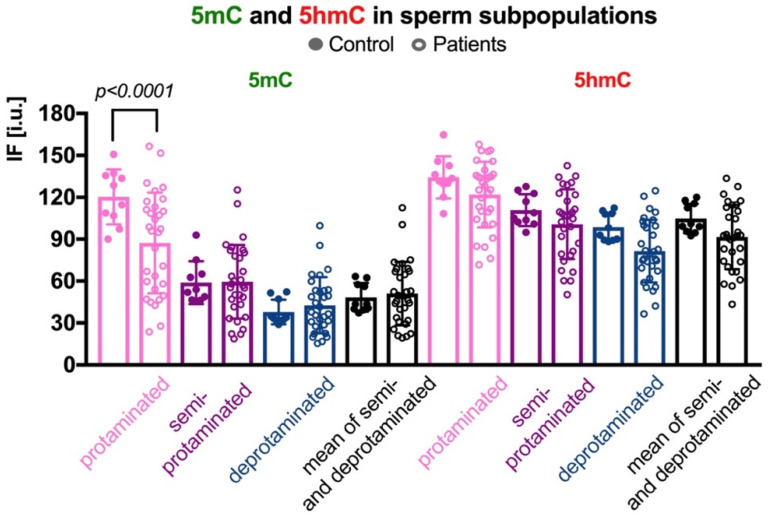
Comparison of mean 5mC and 5hmC DNA levels in three sperm subpopulations, according to the chromatin protamination status in the control vs. patient groups. Each point in the graph represents one case. For each male at least 50 spermatozoa per subpopulation were analysed; bars represent mean values ± SD and all measured points. Subpopulations of spermatozoa are coded with following colour: pink for properly protaminated, purple for semi-protaminated, and blue for deprotaminated ones. Statistical significance was considered at *p* < 0.05.

In three sperm subpopulations defined according to their protamination status, the mean control results for 5mC were as follows: (i) in the correctly protaminated subpopulation: 120.28 ± 19.65 i.u. (range: 90.01–150.67 i.u.; Table 2 and Table S3), which was statistically higher (*p* < 0.0001) than the mean result obtained for the group of patients: 87.29 ± 36.01 i.u. (range: 23.71–156.50 i.u.; Table 2 and Table S3, Figure 3); (ii) in the semiprotaminated subpopulation: mean C value was 58.92 ± 15.41 i.u. (range: 46.30–92.96 i.u.) and was statistically similar to the mean P value: 59.54 ± 26.34 i.u. (range: 18.80–125.35 i.u.); and (iii) in the deprotaminated sperm subpopulation: mean C value: 37.90 ± 8.84 i.u. (range: 28.91–52.25 i.u.) was also similar to the mean P value: 42.73 ± 20.11 i.u. (range: 16.78–99.75 i.u.).

In the case of 5hmC, the mean C and mean P values were similar in all three subpopulations, although some tendency to increase was observed for the C group (Table 2 and Table S3, Figure 3). Results were as follows: (i) in the properly protaminated subpopulation: mean C value 134.26 ± 15.12 i.u. (range: 108.28–164.77 i.u.) vs. mean P value 121.20 ± 23.42 i.u. (range: 71.66–157.83 i.u.); (ii) in the semiprotaminated subpopulation: mean C value 110.85 ± 11.48 i.u. (range: 94.87–127.76 i.u.) vs. mean P value 100.76 ± 24.95 i.u. (range: 50.33–142.54 i.u.); and (iii) in the deprotaminated sperm subpopulation: mean C value 98.71 ± 9.97 i.u. (range: 88.20–112.33 i.u.) vs. mean P value 81.53 ± 22.39 i.u. (range: 36.43–124.70 i.u.).

Also, the 5mC/5hmC ratio was calculated in all the three subpopulations of spermatozoa (Table 2 and Table S3). Statistically significant differences were noted between the two groups of analysed males: (i) in the properly protaminated subpopulation (*p* = 0.0032; mean C: 0.90 ± 0.12, range: 0.66–1.07; mean P: 0.70 ± 0.23, range: 0.33–1.29); and (ii) in deprotaminated subpopulation (*p* = 0.0263; mean C: 0.38 ± 0.07, range: 0.30–0.48; mean P: 0.52 ± 0.19, range: 0.24–1.04). In the subpopulation of semiprotaminated spermatozoa the measured values were similar (*p* > 0.05; mean C: 0.53 ± 0.12, range: 0.42–0.79; mean P: 0.58 ± 0.20, range: 0.31–1.09).

### 2.4. Correlations

**Figure 4:**
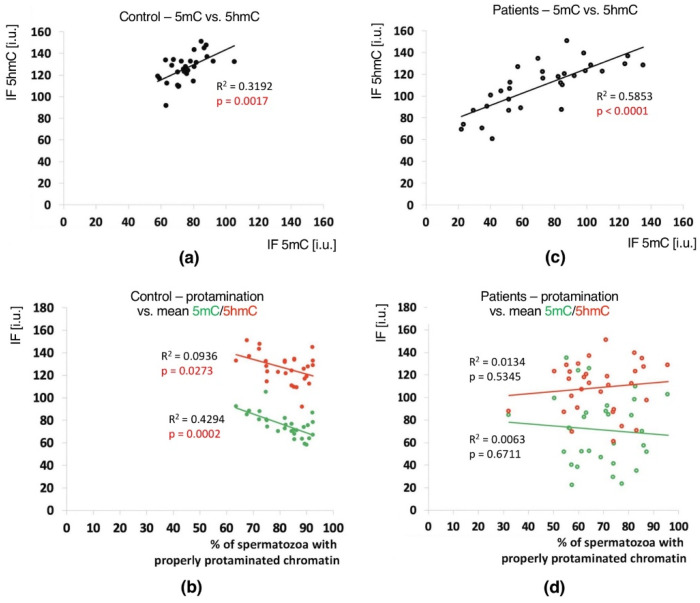
Correlations between global DNA methylation (5mC) and hydroxymethylation (5hmC) levels and sperm chromatin protamination in unfractionated sperm populations in the control C and patient P groups. Each point in the graph represents one case. For each male at least 1500 spermatozoa were used for AB staining, followed by 200 for IF epimark staining. Mean global 5mC vs. mean global 5hmC levels are shown in the control C (**a**) and the patient P groups (**c**). Mean global 5mC and 5hmC levels vs. mean protamination rates are shown in the control C (**b**) and the patient P groups (**d**). Statistical significance was considered at *p* < 0.05.

In the unfractionated total sperm populations, a positive correlation between the mean global 5mC vs. 5hmC values was observed in both groups of males (C: *p* = 0.0017, R^2^ 0.3192, r 0.6667; P: *p* < 0.0001, R^2^ 0.5853, r 0.7340), indicating a clear relationship between these two epimarks (Table 3 and Table S4, Figure 4a,c). In both analysed groups of males, with the increase in methylation level, the level of hydroxymethylation also increased.

**Figure 5:**
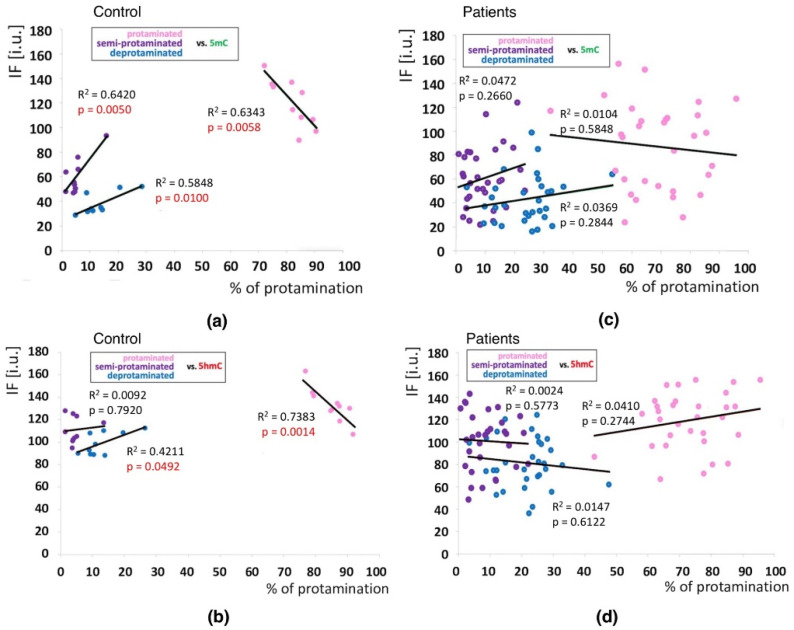
Correlations between methylation (5mC) and hydroxymethylation (5hmC) values (IF) vs. chromatin protamination status shown in three sperm subpopulations in the control and patient groups. (**a**): 5mC vs. protamination in the control group; (**b**): 5hmC vs. protamination in the control group; (**c**): 5mC vs. protamination in the patient group; (**d**): 5hmC vs. protamination in the patient group. Each point in the graph represents one case. For each male at least 50 spermatozoa per subpopulation were analysed. Subpopulations of spermatozoa are coded with following colour: pink for properly protaminated, purple for semi-protaminated, and blue for deprotaminated. Statistical significance was considered at *p* < 0.05.

**Figure 6:**
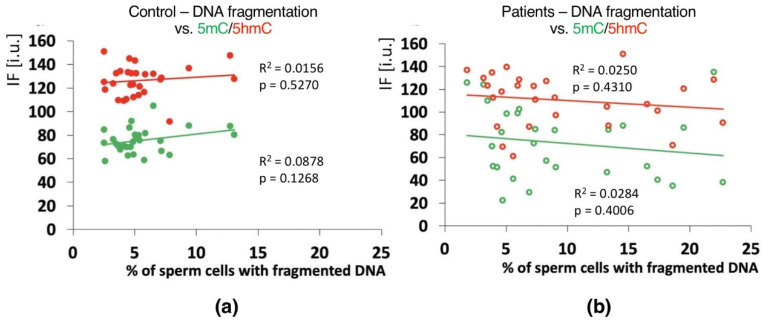
Correlations between global DNA methylation (5mC) and hydroxymethylation (5hmC) vs. mean sperm DNA fragmentation level in unfractionated sperm populations in the control C (**a**) and patient P (**b**) groups. Each point on the graph represents one case. For each male at least 1000 spermatozoa for TUNEL assay and 200 for IF epimark staining were examined. Statistical significance was considered at *p* < 0.05.

Positive correlations between the sperm chromatin protamination status and global 5mC and 5hmC DNA levels were observed in the control group (C) in the unfractionated total sperm population (*p* = 0.0002, R^2^ 0.4294, r −0.6946 for 5mC, *p* = 0.0273, R^2^ 0.0936, r −0.3975 for 5hmC; Figure 4b, Table 3; *p* = 0.0340, R^2^ 0.1615, r −0.4346 for 5mC/5hmC ratio; Table 3 and Table S4), followed by the correlation of particular sperm subpopulations according to their chromatin protamination status (*p* < 0.05; 5mC R^2^ 0.0634, r −0.7746, 5hmC R^2^ 0.7383, r −0.6848; Figure 5a,c Table 3 and Table S4). In the patient group (P), no correlations between 5mC and 5hmC levels and protamination status were observed, either in unfractionated total sperm populations or in the three subpopulations with various chromatin protamination statuses (*p* > 0.05) (Table 3 and Table S4, Figure 4d and Figure 5b,d). Additionally, in patients (P) statistically non-significant opposite tendency was noted: according to increased protamination, 5hmC also rose (Figure 4d and Figure 5d). When considering 5mC vs. protamination in the P group, the correlation was flattened when compared to the C group (Figure 4d and Figure 5c). These observations indicate (i) various levels of epimarks in each of the sperm subpopulations according to chromatin protamination, (ii) a clear correlation between chromatin protamination and 5mC/5hmC levels in normozoospermic controls, and (iii) a loss of correlation in patients with oligo/oligoasthenozoospermia. When evaluating the possible relationship between sperm DNA fragmentation level and 5mC/5hmC levels, no correlations were noted in either group of males (*p* > 0.05; Table 3 and Table S4, Figure 6). Interestingly, the wide distribution of values observed in the P group followed a change in the tendency across the spectrum—from negative to positive (protamination vs. 5hmC) and from positive to negative (DNA fragmentation vs. 5mC and 5hmC), suggesting epigenetic disturbances in these patients (Figure 4b,d and Figure 6b).

Surprisingly, no correlations (*p* > 0.05) were observed in either analysed group of males when collating global 5mC and 5hmC values vs. sperm concentration, total sperm count or ejaculated sample volume, either in unfractionated or subpopulations of sperm (Table 3 and Table S4, Supplementary Figure S1). An inverse tendency (positive in the C group, negative in the P group) in the unfractionated total sperm populations was observed for sperm motility, showing an increase (C) or decrease (P) in 5mC and 5hmC with an increase in motile spermatozoa (Supplementary Figure S1). However, in the P group sperm subpopulations according to protamination status, a statistically significant correlation was observed between total motility and 5hmC (*p* < 0.0001, R^2^ 0.0427, r −0.2067, Table 3 and Table S4). In the C group, there were no statistically significant correlations; however, some tendency (*p* = 0.061) was observed for total motility vs. 5mC (R^2^ 0.1368, r 0.8609) and 5mC/5hmC ratio (R^2^ 0.2653, r 0.8601) (Table 3 and Table S4). Additionally, statistically significant correlations were found in the P group between sperm morphology vs. 5mC (*p* = 0.0216, R^2^ 0.1690, r 0.4111), and/or the 5mC/5hmC ratio (*p* = 0.0096, R^2^ 0.2097, r 0.4579) in the unfractionated total sperm population (Supplementary Figure S1B) and in the sperm protaminated subpopulation (*p* < 0.01; 5mC R^2^ 0.2102, r 0.4585; 5mC/5hmC ratio R^2^ 0.2579, r 0.4883; Table 3 and Table S4). All of these observations may emphasize a weak link between sperm DNA methylation/hydroxymethylation and semen parameters (motility, morphology).

## 3. Discussion

This study is the first to describe the correlation between the particular status of chromatin vs. methylation (5mC) and hydroxymethylation (5hmC) in sperm DNA, as represented by three sperm subpopulations with different protamination status. All analyses were performed sequentially on the same individual sperm cells, meaning that each single spermatozoon, cell by cell, was stained in situ with AB for evaluation of chromatin protamination status, followed by documentation of its position, and then on the same slide subjected to immunofluorescence staining with proper antibodies to detect specific epimarks. Such sequential staining resulted in the generation of unique data comprising a clear relationship between detailed sperm chromatin protamination and global DNA methylation (5mC and 5hmC). Due to technical limitations, the TUNEL assay results have not been included in the sequential staining algorithm (no clear TUNEL signal after AB).

To our knowledge, global methylation analysis of sperm DNA measured via immunofluorescence or colorimetric techniques, followed by microscopy, cytometry or ELISA, has only been described in thirteen previous studies (Supplementary Table S5). In only three studies were the methods based on the chromatographic measurements described [42,79,80]. The majority of published data are based on screening the methylation pattern of particular genes—their promoters or CpGs—using sequencing of single-, few- or whole-genome approaches (Supplementary Table S5). Previous studies have clearly demonstrated a relationship between global 5mC level, sperm quality, sperm apoptosis, abnormal P1/P2 ratio, IVF outcome, age of male patients, and the presence of chromosome aberrations [22,38,42,44,45,63,68,69,70,81]. Additionally, tobacco smoking has been listed as an important external factor disturbing DNA methylation, causing increased sperm DNA damage [82]. Even if the global methylation level is similar between smokers and nonsmokers, an increased variance in methylation patterns, especially in histone-retained regions, was shown in the sperm DNA of smokers [82]. Additionally, alcohol use, obesity, and environmental factors can disrupt the observed DNA methylation patterns of selected genes [41,83,84,85,86]. Interestingly, the role and complexity of epigenetic changes in infertility seem to be underlined by the data documenting alterations in imprinted gene methylation patterns in normozoospermic but infertile males [87,88]. In the present study, the medical history collected for both groups of investigated males (P and C) showed none of the potentially disruptive factors—participants were selected according to questionnaire responses (nonsmokers, no alcohol or drugs, similar age, no toxic agents, normal body mass index (BMI)). Additionally, preparation of tests in situ on slides allowed to be sure not to include into analyses any other cell type that could potentially perturb the data obtained.

Previous studies are incompatible when considering correlations between decreased semen quality (OAT) and protamine content. Correlations between protamine mRNA levels and sperm motility or morphology have been reported [63,89]. However, data suggested only limited trends or showed no linkage, even when levels of methyltransferase mRNA followed by increased DNA methylation were higher [90,91,92,93]. In our study, in the patient group (P) with oligoasthenozoospermia, the frequency of spermatozoa with proper protamination was significantly decreased (−13.46%; *p* < 0.05) compared to normozoospermic controls (Figure 1, Table 1). Additionally, in the control group (C), the total sperm count correlated with the protamination level (*p* < 0.05), but this observation was not relevant in the P group (Table 3). We suggest that the presence of abnormal semen parameters (OA) can be disruptive for correlations found in control samples, although we did not measure the protamine transcript level because of insufficient biological material. However, further studies on isolated protamines according to epimark levels and sperm DNA fragmentation could produce interesting data considering semi-quantitative estimation, especially in the light of a previously documented link between protamine content and DNA fragmentation [94,95,96]. Thus, adding epigenetic data would probably improve the comprehensive interpretation of this phenomenon in the context of infertility.

When considering sperm subpopulations according to their protamination, various levels of 5mC and 5hmC m were documented in each, supported by a clear linkage between chromatin protamination and 5mC/5hmC levels in normozoospermic controls (*p* < 0.01), revealing loss of this correlation in patients with decreased sperm parameters (*p* > 0.05). Additionally, in the properly protaminated sperm subpopulation, 5mC and 5hmC values varied between the P and C groups. The observed lower 5mC and 5hmC values in infertile patients may suggest disturbed spermatogenesis and disrupted maturation of spermatozoa, including improper protamination. This is in agreement with the literature, which shows higher 5mC values in fertile normozoospermic males [70]. It seems to be important, and is shown here for the first time, that there is a heterogeneity of chromatin protamination level within ejaculated sperm, and this can be crucial when considering the global DNA methylation status. This fact is underlined by the observation from unfractionated total sperm populations with similar mean levels of global 5mC and 5hmC observed in both groups of analysed males (P vs. C). Such similarity in global mean values was also documented for other tissue types, which is not unusual since the majority of methylation in the genome occurs in areas outside of CpG islands, such as repetitive elements and noncoding and nonregulatory regions [97,98]. However, attention should be paid to evaluating further obtained mean values to avoid incorrect interpretation, and further analyses should include measurement of specific, detailed parameters, not only general mean values.

Another question is the role of sperm DNA fragmentation (one of the two elements of chromatin integrity measured in this study) and sperm genome methylation changes. In our study, in the control individuals (C), the frequency of spermatozoa with hypermethylated genomes increased with increasing chromatin instability; however, this was represented only by chromatin protamination, not sperm DNA fragmentation (Figure 6). Moreover, in both analysed groups, there was no linkage between sperm DNA fragmentation and semen parameters (Table 3; *p* > 0.05). Global hypermethylation might be an early response to oxidative stress mediated through an increase in DNA methyltransferase (Dnmt) activity [43,99]. Increased hypomethylation following decreased chromatin protamination observed in the patient group suggests a potential association between chromatin structure disturbances and impaired methylation [70,100,101,102,103]. However, we cannot anchor the sperm DNA fragmentation observed in the patient group due to the noted lack of any possible correlations or even tendencies. Thus, it remains questionable whether and how measured sperm DNA fragmentation could be used as one of the fertility parameters. Although some of the literature showed that apoptotic sperm cells (with fragmented DNA) demonstrating hypermethylation of the genome were associated with disorders of spermatogenesis [80,99], the presence of decreased semen parameters themselves cannot be treated as the decisive factor responsible for the observed changes, either in methylation and/or linked to sperm DNA fragmentation. Changes in gene methylation patterns are also well documented in normozoospermic but infertile males [87,88]. However, in a study examining the presence of chromosomal structural aberrations, such correlations were documented [42]. Therefore, when considering sperm DNA fragmentation, attention should be paid to some important aspects, i.e.,: (i) whether the decreased semen parameters resulted from disturbed spermatogenesis, (ii) whether the negative influence of environmental factors led to decreased sperm quality, or (iii) whether the patient had any other abnormalities in karyotype that may influence chromatin stability via the formation of strand breaks resulting from high torsion tensions occurring during the remodelling of sperm chromatin in spermiogenesis. Such tensions may promote sperm chromatin opening and increase apoptosis, leading to a decrease in sperm quality [42,104,105]. Another open question is whether discrimination between single-strand (ss) and double-strand (ds)DNA breaks (i.e., established by COMET assay) would have added value for evaluation of methylation events in a male infertility context. No such data exists to our knowledge). According to the latest literature data available, COMET assay seems to be the most reliable diagnostic method for male infertility, because of its clear indications concerning IVF and ISCI outcomes [106].

Observations of decreased semen parameters linked with sperm DNA global methylation level or altered methylation patterns of imprinted genes have been well documented [66,67,69,70,107,108,109,110,111,112,113,114,115,116]. It was found that in morphologically normal sperm heads, 5mC levels were lower than in abnormal spermatozoa [69,110], and high 5hmC levels were negatively correlated with good sperm head morphology while positively correlated with sperm DNA fragmentation [117]. In asthenozoospermic patients, low motility was linked with sperm DNA hypermethylation [66,69,111,113,118]. In our study, when analysing sperm motility, some interesting tendencies were observed within the total sperm population of unfractionated ejaculated spermatozoa, with a strong correlation (*p* < 0.0001) with the properly protaminated fraction (positive in the C group, negative in the P group), reflecting the decrease in 5mC and 5hmC and a simultaneous increase in motile spermatozoa (Supplementary Figure S1B, Table 3 and Table S4). These observations, supported by the statistically strong correlation noted in the P group (both in the unfractionated and defined sperm subpopulations) between global 5mC and 5mC/5hmC ratio values vs. sperm morphology, apparently linking methylation and hydroxymethylation with sperm motility and morphology. On the other hand, the published data also showed a clear correlation (or suggested trend) between sperm count and/or sperm concentration vs. 5mC level, or possibly no linkage between these two parameters [21,42,66,70,93,108,109,115,116,119,120]. In the present study, both in the unfractionated total sperm samples and in the properly protaminated sperm subpopulation, no correlations were found in either group of males linking global 5mC and 5hmC levels with sperm concentration, total sperm count, or ejaculated sample volume. Such discrepancies can result from various preparations of semen samples, as well as the total number of individuals required for statistical significance. One of the possible influencing factors may be meaningful heterogeneity among semen samples displaying abnormal parameters. Within the same ejaculate, there are several sperm subpopulations that could be fractionated according to chromatin density (gradient centrifugation) or sperm motility (swim-up), and they may reflect different methylation patterns [109,121,122]. Laurentino et al. [122] observed that in OA males there may be a kind of heterogeneity (epimosaicism) among the spermatozoa from one semen sample, revealed as variability in the methylation patterns of selected imprinted genes, probably because of imprint erasure errors. Yu et al. found that in gradient selected sperm fractions, histone retention was decreased, followed by decreased global methylation levels [109]. Interestingly, Dere et al. [123] documented that sperm DNA methylation levels are relatively stable between semen sample collections over a long time period [123]. Additionally, possible DNA from contaminating biological material (when too low attention was given to sample selection/preparation/fractioning), could be a potential cause of incompatible findings across various studies [124,125]. 

In summary, it has been shown here for the first time that there is a heterogeneity within DNA methylation and hydroxymethylation in ejaculated sperm samples according to chromatin protamination status. In OAT patients with a lack of pregnancy success, there was a disruption to the strong correlations between various protamination levels vs. 5mC and 5hmC observed in control normozoospermic males. An interesting linkage was revealed in the relationship of sperm morphology and motility with levels of 5mC and 5hmC, documented in the OAT patient group. Additionally, the wider ranges of values for all studied parameters measured in this group of patients may suggest an association between epigenetic disturbances and decreased semen quality. Following the facts, that proper epimarking and the specific high protamination of sperm chromatin are crucial for correct spermatogenesis, we can suggest that the measurement of the 5mC/5hmC in spermatozoa can be a useful complementary component in the generation of prognostic epidata in cases of male infertility. Questions remain regarding the cause–effect involvement of decreased semen parameters and disturbances in sperm DNA methylation patterns.

## 4. Materials and Methods

**Figure 7:**
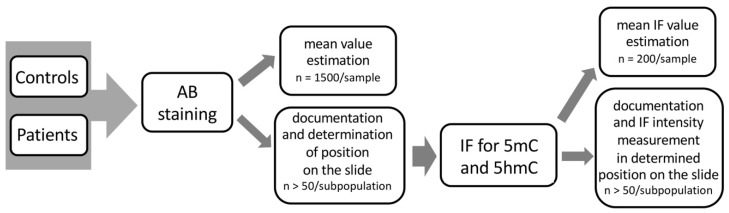
Workflow scheme of the sequential staining algorithm for three sperm subpopulations, including sperm chromatin protamination status and global levels of sperm DNA methylation (5mC) and hydroxymethylation (5hmC).

In this study, correlation was determined between the particular status of sperm chromatin protamination and the global 5mC and 5hmC levels of sperm DNA, using sequential staining of the same spermatozoa: (i) aniline blue staining (AB) to determine three sperm subpopulations depending on their chromatin protamination, followed by documentation of the spermatozoa positions on the slide; and (ii) estimation of global 5mC and 5hmC of sperm DNA on the same spermatozoa with documented protamination (Figure 7). Additionally, the sperm DNA fragmentation level was examined (TUNEL assay) to support the chromatin integrity data. The preparation of tests in situ on slides allowed us also to exclude from analysis any other cell type present in ejaculate that could potentially disrupt the data obtained. All tests were performed for the two analysed groups of males: healthy fertile individuals (C) and patients with oligoasthenozoospermia/oligozoospermia and reproductive failure (P).

### 4.1. Ethics Approval and Consent to Participate

Ethical Committee approval (Local Bioethical Committee at Poznan University of Medical Sciences, approval no. 771/15) was received for the study. All participants were notified about the aim of the study, and provided written informed consent. All experiments were performed in accordance with relevant guidelines and regulations.

### 4.2. Participants

Two experimental groups were included in this study. The control group (C) consisted of 28 healthy males with normozoospermia, proven fertility, and no history of reproductive problems. Ten control donors (C50–C61) were evaluated using a sequential staining algorithm, while for the other 18 C individuals (C5–C33), mean values for particular chromatin parameters were included [42]. The patient group (P) consisted of 31 males with reproductive failure (lack of conception or miscarriages) and oligozoospermia as the main criteria of selection. Each case was screened for karyotype and possible AZF microdeletions. Some patients also revealed decreased parameters for sperm motility and/or morphology (Supplementary Table S1). Males from both groups were selected, with attention given to their similar age (25–30 years), lack of smoking habits, lack of stimulant/drug use, and lack of exposure to toxins in their environment. Ejaculated semen samples were collected after 3–5 days of sexual abstinence. After liquefaction, samples were analysed manually according to the WHO 2010 criteria for semen evaluation (concentration, volume, motility, morphology, and viability) (Supplementary Table S1) [126]. Then, to deplete any traces of seminal plasma, samples were washed in HAM F-10 medium (Gibco; UK), and sperm samples were fixed in a fresh fixative solution (methanol:acetic acid, 3:1 *v*/*v*, −20 °C).

### 4.3. Sperm Chromatin Integrity

Sperm chromatin integrity status was evaluated via two tests: aniline blue (AB) staining for determination of sperm chromatin protamination, and TUNEL assay for determination of sperm DNA fragmentation level.

#### 4.3.1. Sperm Chromatin Protamination Status

Sperm chromatin protamination status was evaluated using aniline blue (AB) staining [127]. Aniline blue is a reagent that binds to lysine residues in histones, resulting in dark blue staining and allowing us to determine the protamines:histones proportion. Slides with fixed sperm cells were washed (methanol:acetic acid, 3:1 *v*/*v*, −20 °C) in 2× SSC (3 min) and air dried, and then 100 µL of 1% eosin-Y solution (Merck; Germany) was applied onto slides for 3 min at RT and rinsed off with water. Slides were then stained in acidic 5% aniline blue solution (Water Blue, Fluka; Germany) for 5 min, rinsed off, air-dried, and analysed using a light microscope (Olympus BX40, Japan; oil immerse objective 100×). After AB staining, three subpopulations of spermatozoa could be recognised: pink—sperm cells with proper protamine to histone ratio, purple-pink—spermatozoa with disturbed protamines:histones ratio, and navy blue—deprotaminated sperm cells with a high proportion of remaining histones (Figure 1b). In each sample (all males from C and P groups), approximately 1500 spermatozoa were examined, followed by documentation of at least 50 spermatozoa per coloured subpopulation for further immunofluorescence staining with epimarks. Statistical power calculation revealed that the number of analysed cells should be minimum 306 for AB staining, and 45 for IF counting, in respective sperm subpopulations. Image documentation was performed using CellSense Dimension software (ver. 1.14, Olympus, Germany) and included determination of particular sperm cell localization on slides, using coordinate values (XY) depicted on the rulers at the microscopic stage.

#### 4.3.2. Sperm DNA Fragmentation

The sperm DNA fragmentation level was estimated using the TUNEL assay on slides (Flow TACS Apoptosis Detection Kit, R & D Systems, Minneapolis, MN, USA), which allows for identification of sperm cells with fragmented DNA [128]. The principle of the technique involves complex formation between biotinylated DNA fragments and streptavidin-conjugated fluorescein (FITC) in the presence of terminal deoxynucleotidyl transferase (TdT). Two populations of sperm cells can be recognized: fluorescence-labelled TUNEL-positive cells (fragmented DNA, light green) and TUNEL-negative cells labelled only with DAPI (nonfragmented DNA, blue). In each case (all individuals from the C group and 27/31 out of P group (for 4 patients there was not enough biological material for evaluation), at least 1000 sperm cells (power calculation value = 278) were counted using a fluorescence microscope (Leica DM5500, equipped with 100× oil immersion objective and SpO/FITC/Triple/DAPI filters). TUNEL assay was performed on the separate slides (not used for AB and/or IF stainings). Thus, the results of TUNEL assay refer to the mean global values for the whole ejaculate samples (without separation into sperm subpopulations). The TUNEL assay was selected as the method for sperm DNA fragmentation evaluation because of the fact that among the variety of available techniques, it is routinely used, reproducible, and the sperm chromatin integrity remains intact, which is important for other experimental approaches concerning e.g., sperm nuclear order [129,130,131]. 

### 4.4. Immunofluorescence (IF)

Immunofluorescence in situ was used to detect and measure the epimarks for global sperm DNA methylation (5mC) and hydroxymethylation (5hmC). This method has been validated previously when correlated to thin-layer chromatography (TLC) [42]. Specific antibodies conjugated to fluorochromes were applied: primary antibodies—mouse anti-5mC 1:200 (clone 33D3, cat no. MABE146, Merck), and rat anti-5hmC 1:1000 (cat no. ab106918, Abcam); secondary antibodies—goat anti-mouse-FITC 1:400 (cat no. F2012, Sigma-Aldrich), and goat anti-rat-AF594 1:800 (cat no. ab150160, Abcam). Antibodies were diluted in 1%BSA/1× PBST. Two negative controls were performed for each series of experiments: (i) without primary antibody to check the specificity of the binding, and (ii) without secondary antibody to check the possible fluorescence background level. First, samples with fixed sperm smears after AB staining and documentation were destained in 100% xylene reagent, followed by a series of washes in 1× PBST (pH 7.4, room temp., 5 min. each). Xylene washing is required for removal of any traces of oil immersion and aniline blue stain, and does not influence the immunofluorescent signals. Then, slides were incubated in 25 mM DTT/1 M Tris-HCl, pH 9.5, at room temperature for 20 min for slight decondensation of the chromatin. The degree of decondensation was fully controlled: only spermatozoa with an unaffected tail, preserved sperm head shape and a decondensed nucleus size no larger than 1.4-fold were selected. A series of washes in 1× PBST was followed by incubation in 6 N HCl. Then, blocking with 1% BSA/1× PBST for 30 min was performed. Next, overnight incubation with a mix of primary anti-m5C and anti-5hmC antibodies was performed in a humidified container at 37 °C. After double washing the samples with 1× PBST, secondary antibodies conjugated to selected fluorochromes (FITC or AF594; 60 min) were applied. Next, unconjugated antibodies were washed out (4× in 1× PBST, 5 min each). For the final detection, 20 mL of DAPI/Vectashield (Sigma-Aldrich) was added to the samples, and further analysis was performed. Approximately 200 sperm cells in each case (unfractionated into subpopulations; power calculation value = 132) were evaluated, followed by documentation and analysis of at least 50 sperm cells (power calculation value = 45) per chromatin status within the studied sperm subpopulation. Images of the IF results were acquired using a fluorescence microscope with a suitable filter set, and CellSense Dimension (Olympus) software was used (Olympus BX40, Japan; filters: DAPI/FITC/SpO/Triple; objectives: 10× and 100× with oil immersion; CCD camera). Measurements of the 5mC and 5hmC signal intensity (i.u.—intensity unit) were performed using CellSense Dimension software in-built measurement tools (‘Measure’ > ‘Intensity Profile’). The intensity of fluorescence (cell fluorescence, IF; international unit, i.u.) was calculated for each spermatozoon, including the integrated density, area of the sperm cell nucleus, and correction of the background fluorescence (measured in 5 areas outside of the spermatozoa containing only dark segments). The workflow scheme concerning the measurement of fluorescence is shown in Supplementary Figure S2. The total number of spermatozoa evaluated in the study amounted to approximately 171,100 counted (nondifferentiated) and 6150 cells documented in three analysed subpopulations.

### 4.5. Statistical Analysis

Statistical analyses for each parameter included normality testing (D’Agostino-Pearson), ANOVA, and Fisher’s exact test for determination of differences between mean values, followed by Bonferroni correction, two-tailed Pearson correlation, and linear regression analysis for determination of possible correlations between evaluated parameters. All tests were performed with a significance level of α = 0.05 using GraphPad Prism (v.7.0e) or Analyze-it for Excel (v. 5.11) software. Statistical power calculation was performed using an online Sample Size Calculator (http://www.raosoft.com/samplesize.html, accessed on 10 March 2018) to determine the minimum number of analysed cells that should be assessed for a particular test (with standard assumptions: 95% confidence level, 5% error, 50% population).

## Figures and Tables

**Table 1:** Characteristics of sperm chromatin parameters and levels of DNA epimarks in the unfractionated sperm population obtained from a group of 28 control individuals (C) and 31 infertile patients (P). The results for sperm chromatin integrity concern sperm chromatin deprotamination (AB) and sperm DNA fragmentation (TUNEL), while sperm DNA epimarks’ level was determined for global sperm DNA methylation (5mC) and hydroxymethylation (5hmC) (IF—immunofluorescence). The analyses were performed for all males from C and P groups, with the exception of the TUNEL assay which was applied for 27/31 males out of P group (for 4 males there was not enough biological material).

			Control Group: Mean C ± *SD*	Patients’ Group: Mean P ± *SD*
**SPERM CHROMATIN** **INTEGRITY**	**sperm chromatin** **protamination** **[%; aniline blue** **assay, AB]**	**protaminated [pink]**	82.02 ± *8.31*	68.56 * ± *13.59*
		**semiprotaminated** **[purple]**	5.17 ± *4.21*	9.86 ± *6.68*
		**deprotaminated** **[navy blue]**	12.82 ± *6.86*	21.59 * ± *8.82*
		**sum of semi- and** **deprotaminated**	17.98 ± *8.31*	31.44 * ± *13.59*
	**spermatozoa with fragmented DNA** **[%; TUNEL assay]**		5.52 ± *2.62*	9.55 ± *6.29*
**EPI-MARKS**	**in unfractionated total sperm** **population**	**mean 5mC [IF i.u.]**	75.61 ± *10.66*	71.32 ± *30.82*
		**mean 5hmC [IF i.u.]**	126.33 ± *13.17*	109.03 ± *22.74*
		**5mC/5hmC ratio**	0.60 ± *0.07*	0.64 ± *0.20*

Mean P-values statistically significant (*p* < 0.05) from the mean C values are marked with *.

**Table 2:** Results of 5mC and 5hmC measurements in three subpopulations of spermatozoa fractionated according to their chromatin protamination status.

	Subpopulation	Control Group: Mean C ± *SD*	Patients’ Group: Mean P ± *SD*
**5mC [IF i.u.]**	**protaminated [pink]**	120.28 ± *19.65*	87.29 * ± *36.01*
	**semiprotaminated [purple]**	58.92 ± *15.41*	59.54 ± *26.34*
	**deprotaminated [navy blue]**	37.90 ± *8.84*	42.73 ± *20.11*
	**mean of semi- and deprotaminated**	48.41 ± *10.23*	51.13 ± *22.72*
**5hmC [IF i.u.]**	**protaminated [pink]**	134.26 ± *15.12*	121.20 ± *23.42*
	**semiprotaminated [purple]**	110.85 ± *11.48*	100.76 ± *24.95*
	**deprotaminated [navy blue]**	98.71 ± *9.97*	81.53 ± *22.39*
	**mean of semi- and deprotaminated**	104.78 ± *10.51*	91.15 ± *23.03*
**5mC/5hmC ratio**	**protaminated [pink]**	0.90 ± *0.12*	0.70 ** ± *0.23*
	**semiprotaminated [purple]**	0.53 ± *0.12*	0.58 ± *0.20*
	**deprotaminated [navy blue]**	0.38 ± *0.07*	0.52 * ± *0.19*
	**mean of semi- and** **deprotaminated**	0.46 ± *0.07*	0.55 ± *0.19*

Mean P-values statistically significant (*p* < 0.05) from mean C values are marked with * for *p* < 0.05, and ** for *p* < 0.01; at least 50 spermatozoa were analysed per population in each case.

**Table 3:** Analysis of correlations between all analysed parameters in Control and Patient groups.

		Control Group (C)	Sperm Chromatin Integrity [%]		Unfractionated Total Sperm Population			Properly Protaminated Subpopulation			Semen Parameters					
Patients’ Group (P)			Protaminated	Fragmented DNA	Glo-bal 5mC	Glo-bal 5hmC	5mC/5hmC Ratio	Mean 5mC	Mean 5hmC	5mC/5hmC Ratio	Concen-tration [×10^6^/mL]	Volume [mL]	Total Count [×10^6^]	Morphology [%]	Motility [%]	
															Progres-sive	Total
**Sperm Chromatin Integrity [%]**		**Protaminated**		ns	**	*	*	**	**	ns	ns	ns	*	ns	ns	ns
		**Fragmented DNA**	ns		ns	ns	ns	ns	ns	ns	ns	ns	ns	ns	ns	ns
**Unfractionated Total Sperm Population**		**Global 5mC**	ns	ns		**	***	ns	ns	ns	ns	ns	#ns	ns	ns	ns
		**Global 5hmC**	ns	ns	***		ns	**	ns	*	ns	ns	ns	ns	ns	ns
		**5mC/5hmC Ratio**	ns	ns	***	**		ns	ns	ns	ns	ns	ns	ns	ns	ns
**Properly Protaminated Subpopulation**		**Mean 5mC**	ns	ns	***	***	***		#ns	*	ns	ns	ns	ns	ns	#ns
		**Mean 5hmC**	ns	ns	***	***	**	***		ns	#ns	ns	ns	ns	ns	ns
		**5mC/5hmC Ratio**	ns	ns	***	**	***	***	*		ns	ns	ns	ns	ns	#ns
**Semen Parameters**	**Concentration [×10^6^/mL]**		ns	ns	ns	ns	ns	ns	ns	ns		*	***	ns	*	*
	**Volume [mL]**		ns	ns	ns	ns	ns	ns	ns	ns	ns		ns	ns	ns	ns
	**Total Count [×10^6^]**		ns	ns	ns	ns	ns	ns	ns	ns	***	**		ns	ns	ns
	**Morphology [%]**		ns	ns	*	ns	**	**	ns	**	ns	ns	#ns		*	**
	**Motility [%]**	**Progressive**	ns	ns	ns	ns	ns	ns	ns	ns	ns	ns	ns	ns		**
		**Total**	ns	ns	ns	ns	ns	ns	***	ns	ns	ns	ns	ns	***	

Statistical description: *** *p* < 0.0001, ** 0.0001 < *p* < 0.01, * 0.01 < *p* < 0.05, ns—statistically non-significant (*p* > 0.05), #ns 0.05 < *p* < 0.06 (statistically non-significant but at the border value); grey colour—values statistically significant.

## Data Availability

All the data generated or analysed during this study are included in this published article (and its Supplementary Information Files).

## Supplementary Materials

The following supporting information can be downloaded at: https://www.mdpi.com/article/10.3390/ijms23094516/s1. Reference [132] is cited in the supplementary materials.
