# Supplementary material for: Global 5mC and 5hmC DNA Levels in Human Sperm Subpopulations with Differentially Protaminated Chromatin in Normo- and Oligoasthenozoospermic Males
Source: Int J Mol Sci. 2022 Apr 19;23(9):4516. doi: 10.3390/ijms23094516 (PMC9099774; doi:10.3390/ijms23094516)
Supplement: Supplementary file 1 [file ijms-23-04516-s001.zip › ijms-1681883-supplementary.pdf]

Global 5mC and 5hmC DNA levels in human sperm subpopulations with differentially protaminated chromatin in normo- and oligoasthenozoospermic males

Marta Olszewska[1*], Oliwia Kordyl[2], Marzena Kamieniczna[1], Monika Fraczek[1], Piotr Jedrzejczak[3], Maciej Kurpisz[1*]

[1] Institute of Human Genetics, Polish Academy of Sciences, Strzeszynska 32, 60-479 Poznan, Poland; marta.olszewska@igcz.poznan.pl (M.O.), marzena.kamieniczna@igcz.poznan.pl (M.Ka.), monika.fraczek@igcz.poznan.pl (M.F.), maciej.kurpisz@igcz.poznan.pl (M.Ku.)
[2] Faculty of Biology, Adam Mickiewicz University in Poznan, 61-614 Poznan, Poland; oliwiakordyl@gmail.com (O.K.)
[3] Division of Infertility and Reproductive Endocrinology, Department of Gynecology, Obstetrics and Gynecological Oncology, Poznan University of Medical Sciences, 60-535 Poznan, Poland; piotrjedrzejczak@gmail.com (P.J.)
* Correspondence: marta.olszewska@igcz.poznan.pl (M.O.), maciej.kurpisz@igcz.poznan.pl (M.Ku.)

ORCID IDs:

Marta Olszewska	0000-0002-2337-4545
Oliwia Kordyl	0000-0001-6139-8276
Marzena Kamieniczna	0000-0002-9619-8220
Monika Fraczek	0000-0003-1212-4230
Piotr Jedrzejczak	0000-0003-2437-7737
Maciej Kurpisz	0000-0003-3275-3245

Figure S1 Correlations between global sperm DNA methylation (5mC) and hydroxymethylation (5hmC) vs. sperm semen parameters in the Control group (**A**) and the group of Patients (**B**). Unfractionated sperm samples were analyzed.

Figure S2 Schematic workflow and technical details concerning quantification of the fluorescent signal in immunofluorescence staining procedure. **A** Technical parameters required for proper signal acquisition using Olympus BX40 microscope and CellSense Dimension software (Olympus), performed on control samples from normozoospermic, fertile donor; **B** schematic representation of control slides used for determination of potential differences in immunofluorescent signal detection according to the presence of aniline blue (AB) stain for chromatin protamination status and/or single or mixed usage of antibodies specific for methylation (5mC) and hydroxymethylation (5hmC) of sperm DNA; **C** schematic flow chart displaying basic steps of the fluorescence intensity measurement via CellSense Dimension software, according to manufacturers' manual (Olympus).

Table S1 Characteristics of semen parameters in Control (C) and Patients' (P) groups (according to the WHO guidelines, 2010). Mean P values statistically significant ($p<0.05$) from mean C values were marked with *.

Table S2 Characteristics of sperm DNA obtained from a group of 28 control individuals (C) and 31 patients (P). C50-C61: control individuals analysed further according to sperm chromatin status; C5-C33: control individuals analysed only for mean values in unfractionated total sperm samples. The results for sperm chromatin integrity (sperm chromatin deprotamination (AB) and sperm DNA fragmentation (TUNEL)), and global sperm DNA methylation (5mC) and hydroxymethylation (5hmC) levels in the unfractionated sperm population (IF) are presented. The analyzes were performed for all males from C and P groups, with exception of TUNEL assay with 27/31 out of P group (for 4 males there was not sufficient biological material). Mean P-values statistically significant ($p<0.05$) from the mean C values are marked with *, n = total number of spermatozoa counted in each case per method, nd = not done.

Table S3 Results of 5mC and 5hmC measurements in three subpopulations of spermatozoa fractionated with respect to their chromatin protamination status. Mean P-values statistically significant ($p<0.05$) from mean C values are marked with * for $p<0.05$, and ** for $p<0.01$. n = total number of spermatozoa analyzed in each case (at least 50 per population).

Table S4 Analysis of correlations between all analyzed parameters in Control and Patients' groups. Statistical description: *** $p<0.0001$, ** $0.0001<p<0.01$,* $<0.01p<0.05$, ns - statistically non-significant ($p>0.05$), #ns $0.05<p<0.06$ (statistically non-significant but at the border value). Correlation coefficients: R^2 for linear regression and Pearson *r* values included for statistically significant and border results.

Table S5 Literature data concerning methylation analysis of sperm DNA in fertile and infertile males. N – normozoospermia, O – oligozoospermia, A – asthenozoospermia, T – teratozoospermią, IVF – in vitro fertilization, ICSI – intracytoplasmic sperm injection, P1, P2 – protamine 1, 2, IF – immunofluorescence, IHC – immunohistochemistry, ELISA - enzyme-linked immunosorbent assay, HPLC – high pressure liquid chromatography, TLC – thin-layer chromatography, UPLC-MS/MS – ultra performance liquid chromatography - tandem mass spectrometry

Figure S1 Correlations between global sperm DNA methylation (5mC) and hydroxymethylation (5hmC) vs. sperm semen parameters in the Control group (**A**) and the group of Patients (**B**). Unfractionated sperm samples were analyzed.

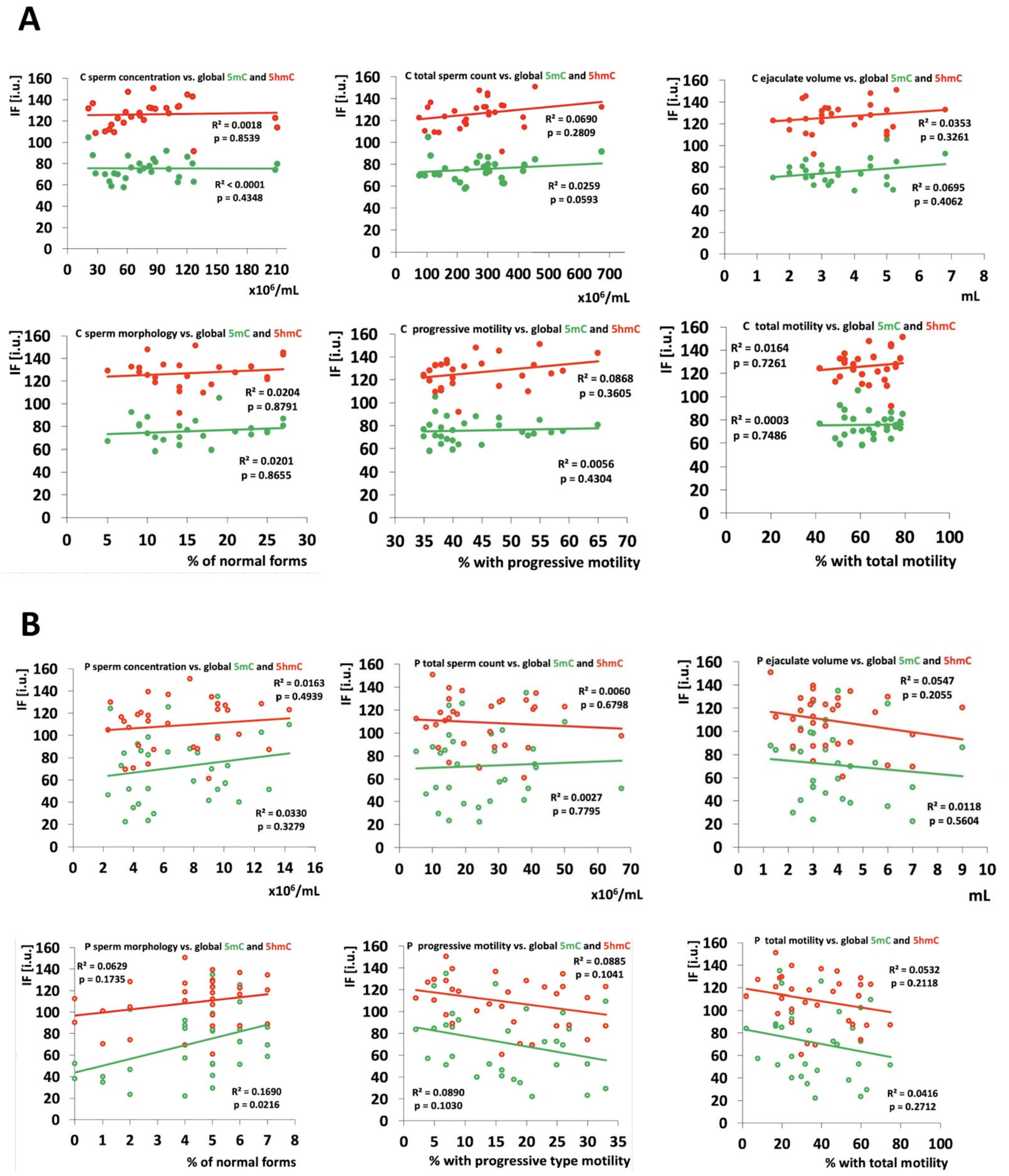

Figure S2 Schematic workflow and technical details concerning quantification of the fluorescent signal in immunofluorescence staining procedure. **A** Technical parameters required for proper signal acquisition using Olympus BX40 microscope and CellSense Dimension software (Olympus), performed on control samples from normozoospermic, fertile donor; **B** schematic representation of control slides used for determination of potential differences in immunofluorescent signal detection according to the presence of aniline blue (AB) stain for chromatin protamination status and/or single or mixed usage of antibodies specific for methylation (5mC) and hydroxymethylation (5hmC) of sperm DNA; **C** schematic flow chart displaying basic steps of the fluorescence intensity measurement via CellSense Dimension software, according to manufacturers' manual (Olympus).

A

parameter	final setting
determined constant value of control sample with non overexposed fluorescent signal; checked everytime for each series of slides	
light intensity constant through an experiment<br>lack of overexposure of evaluated sample (proper reduction of the exposure's time to the constant value)<br>reduction of the noise from background (settings of camera signal amplification and exposure time)	Constant intensity of the fluorescent burner in used microscope<br>Constant ISO value = 400<br>Constant time of exposure = 1 s<br>Exposure compensation value = 0
reduction of the background fluorescence	5 areas outside the spermatozoa containing only dark segments measured for the background correction with 'Background Substraction' option

B

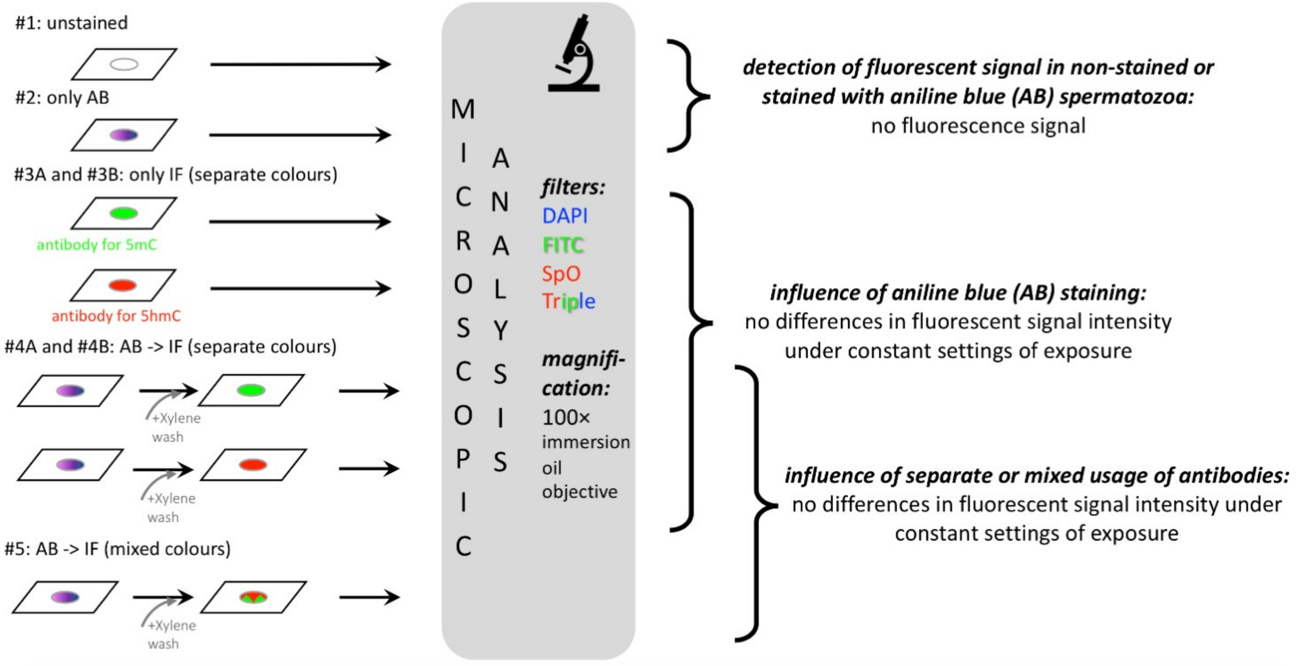

C

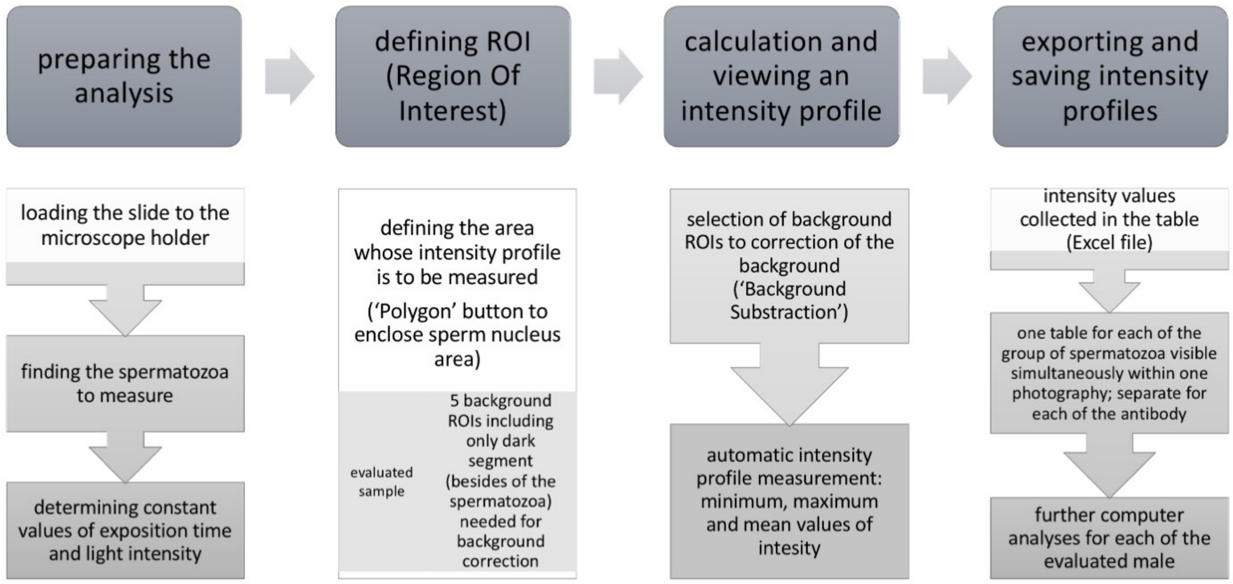

Table S1 Characteristics of seminological parameters in Control (C) and Patients' (P) groups (according to the WHO guidelines, 2010). Mean P values statistically significant (p<0.05) from mean C values were marked with *.

	Control group n=28	concentration [x106/mL]	volume [mL]	total count [x106]	% of morphologically normal forms	motility [%]	
						progressive	total
fractionated into three subpopulations	**C50**	20,70	5,00	**103,50**	19,00	37,00	59,00
	**C52**	37,30	2,50	**93,25**	14,00	38,00	61,00
	**C53**	101,23	3,00	**303,69**	21,00	59,00	76,00
	**C55**	125,00	2,40	**300,00**	27,00	65,00	74,00
	**C56**	110,30	3,20	**352,96**	14,00	45,00	66,00
	**C57**	84,00	3,50	**294,00**	23,00	54,00	78,00
	**C58**	208,00	2,00	**416,00**	25,00	52,00	72,00
	**C59**	46,40	2,70	**125,28**	17,00	53,00	64,00
	**C60**	44,00	5,20	**228,80**	18,00	40,00	51,00
	**C61**	76,50	3,00	**229,50**	25,00	40,00	68,00
unfractionated total sperm population	**C5**	210,00	2,00	**420,00**	14,00	48,00	71,00
	**C7**	82,00	3,50	**287,00**	23,00	38,00	78,00
	**C8**	60,60	4,50	**272,70**	10,00	44,00	64,00
	**C9**	126,30	2,75	**347,33**	14,00	41,00	74,00
	**C10**	120,00	2,50	**300,00**	27,00	48,00	74,00
	**C11**	99,00	6,80	**673,20**	8,00	37,00	51,00
	**C13**	50,30	1,50	**75,45**	11,00	35,00	57,00
	**C14**	72,00	4,50	**324,00**	9,00	36,00	57,00
	**C15**	87,80	3,00	**263,40**	9,00	42,00	53,00
	**C21**	58,00	3,30	**191,40**	5,00	40,00	53,00
	**C24**	41,70	5,00	**208,50**	nd	38,00	49,00
	**C27**	63,70	2,50	**159,25**	15,00	35,00	42,00
	**C28**	85,90	5,30	**455,27**	16,00	55,00	79,00
	**C29**	25,00	4,50	**112,50**	nd	39,00	53,00
	**C30**	28,00	5,00	**140,00**	nd	37,00	72,00
	**C31**	72,50	4,20	**304,50**	10,00	57,00	76,00
	**C32**	111,50	3,10	**345,65**	12,00	39,00	66,00
	**C33**	56,00	4,00	**224,00**	11,00	36,00	61,00
	**mean C**	**82,28**	**3,59**	**269,68**	**15,88**	**43,86**	**64,25**
	SD	***46,91***	***1,25***	***129,73***	***6,39***	***8,37***	***10,46***
	**Patients' group n=31**						
fractionated into three subpopulations	**P2**	5,00	3,00	**15,00**	5,00	8,00	25,00
	**P3**	12,50	2,50	**31,25**	2,00	20,00	60,00
	**P12**	7,75	1,30	**10,08**	4,00	7,00	17,00
	**P14**	2,50	6,00	**15,00**	5,00	5,00	20,00
	**P18**	6,30	2,20	**13,86**	4,00	5,00	20,00
	**P19**	3,20	5,50	**17,60**	6,00	25,00	46,00
	**P22**	4,30	3,80	**16,34**	4,00	9,00	25,00
	**P24**	10,10	3,00	**30,30**	4,00	4,00	8,00
	**P52**	6,33	3,00	**18,99**	6,00	15,00	40,00
	**P65**	11,00	2,50	**27,50**	1,00	12,00	25,00
	**P67**	5,00	2,50	**12,50**	5,00	17,00	34,00
	**P72**	4,00	6,00	**24,00**	1,00	19,00	33,00
	**P73**	8,25	3,50	**28,88**	6,00	27,00	56,00

**P75**	4,50	9,00	**40,50**	7,00	8,00	17,00
**P77**	9,60	4,00	**38,40**	5,00	7,00	19,00
**P88**	3,40	1,50	**5,10**	5,00	2,00	2,00
**P90**	9,60	7,00	**67,20**	5,00	7,00	18,00
**P110**	13,00	3,00	**39,00**	6,00	25,00	75,00
**P111**	3,70	3,00	**11,10**	5,00	14,00	32,00
**P112**	10,25	4,00	**41,00**	6,00	23,00	60,00
**P113**	9,00	4,20	**37,80**	5,00	16,00	30,00
**P125**	5,00	3,00	**15,00**	2,00	30,00	60,00
**P141**	2,32	3,50	**8,12**	2,00	16,00	38,00
**P146**	3,47	7,00	**24,29**	4,00	21,00	37,00
**P151**	5,00	3,00	**15,00**	0,00	30,00	59,00
**P155**	4,35	4,50	**19,58**	0,00	18,00	54,00
**P221**	14,30	3,50	**50,05**	6,00	33,00	65,00
**P228**	9,60	2,90	**27,84**	5,00	26,00	49,00
**P229**	5,36	2,20	**11,79**	5,00	33,00	63,00
**P233**	9,20	4,50	**41,40**	7,00	26,00	48,00
**P246**	8,00	4,00	**32,00**	7,00	8,00	25,00
**mean P**	**6,96***	**3,83**	**25,37***	**4,35***	**16,65***	**37,42***
SD	***3,32***	***1,72***	***14,15***	***1,99***	***9,37***	***18,96***

Table S2. Characteristics of sperm DNA obtained from a group of 28 control individuals (C) and 31 patients (P). C50-C61: control individuals analysed further according to sperm chromatin status; C5-C33: control individuals analysed only for mean values in unfractionated total sperm samples. The results for sperm chromatin integrity (sperm chromatin deprotamination (AB) and sperm DNA fragmentation (TUNEL)), and global sperm DNA methylation (5mC) and hydroxymethylation (5hmC) levels in the unfractionated sperm population (IF) are presented. The analyzes were performed for all males from C and P groups, with exception of TUNEL assay with 27/31 males out of P group (for 4 males there was not sufficient biological material). Mean P-values statistically significant ($p<0.05$) from the mean C values are marked with *, n = total number of spermatozoa counted in each case per method, nd = not done.

	sperm chromatin integrity							epimarks			
	sperm chromatin protamination [%; aniline blue assay]					spermatozoa with fragmented DNA [TUNEL assay]		in unfractionated total sperm population			
**Control group (C) n=28**	**protaminated [pink]**	**semi-protaminated [purple]**	**deprotaminated [navy blue]**	**sum of semi- and deprotaminated**	**n**	**%**	**n**	**mean 5mC [IF i.u.]**	**mean 5hmC [IF i.u.]**	**n**	**5mC/5hmC ratio**
**C50**	74.80	5.71	19.49	25.20	1488	6.50	1000	105.06	132.18	246	0.79
**C52**	84.46	4.67	10.87	15.54	1499	4.33	1200	70.22	110.52	233	0.64
**C53**	90.60	4.03	5.37	9.40	1490	7.10	1000	75.12	127.55	228	0.59
**C55**	72.16	1.47	26.37	27.84	1520	5.04	1151	80.37	143.25	267	0.56
**C56**	85.50	5.60	8.90	14.50	2000	4.43	1329	62.95	133.66	255	0.47
**C57**	81.96	4.17	13.87	18.04	2016	3.50	1000	72.54	132.55	232	0.55
**C58**	75.32	15.52	9.17	24.69	2204	4.85	1133	74.22	123.14	247	0.60
**C59**	85.30	4.36	10.34	14.70	2708	3.65	1095	71.12	109.55	282	0.65
**C60**	89.39	1.34	9.27	10.61	2244	5.78	1156	59.00	116.76	215	0.51
**C61**	82.18	4.34	13.48	17.82	2166	5.40	1000	75.90	121.30	233	0.63
**C5**	75.10	16.60	8.30	24.90	1501	5.32	1109	79.91	114.28	209	0.70
**C7**	92.35	3.03	4.62	7.65	1781	5.11	1272	78.11	132.78	218	0.59
**C8**	72.42	5.49	22.09	27.58	1549	12.73	1092	87.92	147.71	255	0.60
**C9**	88.27	0.90	10.83	11.73	1475	7.84	1148	63.25	91.71	240	0.69
**C10**	92.16	1.47	6.37	7.84	2015	4.58	1178	86.53	144.90	211	0.60
**C11**	63.60	14.54	21.86	36.40	2000	4.73	1205	92.14	132.54	231	0.70
**C13**	79.29	2.86	17.85	20.71	1855	4.65	1032	70.14	122.75	219	0.57
**C14**	75.00	1.47	23.53	25.00	1468	13.06	1141	80.38	127.80	215	0.63
**C15**	81.83	4.23	13.94	18.17	1497	5.83	1012	81.72	131.57	203	0.62
**C21**	92.37	1.91	5.72	7.63	1574	7.15	1007	66.76	128.86	214	0.52
**C24**	91.10	1.27	7.63	8.90	1561	4.92	1057	63.52	112.44	229	0.56
**C27**	84.44	4.67	10.89	15.56	1644	3.24	1080	76.58	124.06	230	0.62
**C28**	67.70	5.70	26.60	32.30	1502	2.50	1000	84.82	150.98	228	0.56

**C29**	68.45	9.71	21.84	31.55	1586	9.37	1014	88.34	136.93	216	0.65
**C30**	86.27	4.48	9.25	13.73	1557	4.10	1098	70.89	109.16	215	0.65
**C31**	88.98	7.60	3.42	11.02	1662	2.50	1000	73.71	125.31	205	0.59
**C32**	85.50	5.58	8.92	14.50	2000	3.81	1050	67.78	134.21	277	0.51
**C33**	90.04	1.91	8.05	9.96	1564	2.60	1000	57.97	118.83	236	0.49
**mean C**	**82.02**	**5.17**	**12.82**	**17.98**	**1754.50**	**5.52**	**1106.40**	**75.61**	**126.33**	**243.80**	**0.60**
SD	***8.31***	***4.21***	***6.86***	***8.31***	***313.94***	***2.62***	***109.99***	***10.66***	***13.17***	***19.97***	***0.07***
**Patients' group (P) n=31**											
**P2**	82.61	2.57	14.82	17.39	2490	5.04	1250	98.25	139.35	203	0.71
**P3**	95.74	0.80	3.46	4.26	2744	6.07	1005	102.52	128.50	288	0.80
**P12**	71.06	4.01	24.93	28.94	1546	14.52	1019	87.78	150.80	220	0.58
**P14**	60.01	13.75	26.24	39.99	2298	3.15	1016	123.92	129.69	250	0.96
**P18**	72.12	6.99	20.88	27.87	1559	7.36	1019	84.87	110.59	244	0.77
**P19**	56.32	16.02	27.66	43.68	2148	nd	-	72.73	116.45	209	0.62
**P22**	71.69	2.91	25.39	28.30	1681	nd	-	92.36	118.63	210	0.78
**P24**	86.20	4.88	8.93	13.81	1640	8.27	1016	57.22	127.02	215	0.45
**P52**	64.40	10.40	25.20	35.60	1529	1.81	1050	125.60	136.85	233	0.92
**P65**	57.35	17.54	25.11	42.65	2406	17.36	1152	40.21	100.99	217	0.40
**P67**	62.43	22.57	14.99	37.56	2144	4.62	1039	82.33	117.96	208	0.70
**P72**	83.45	4.56	11.99	16.55	2026	18.60	1011	34.96	70.58	200	0.50
**P73**	32.28	20.03	47.69	67.72	1597	13.34	1012	84.30	87.70	210	0.96
**P75**	63.29	11.04	25.68	36.72	1540	19.50	1631	86.07	120.51	218	0.71
**P77**	55.37	21.39	23.23	44.62	2024	21.94	1094	134.98	128.51	226	1.05
**P88**	81.33	7.49	11.18	18.67	2162	8.96	1038	83.88	112.36	230	0.75
**P90**	87.40	3.87	8.73	12.60	1810	9.05	1050	51.55	97.32	234	0.53
**P110**	54.33	24.11	21.56	45.67	1406	4.26	1033	51.56	87.09	241	0.59
**P111**	61.35	9.33	29.32	38.65	2064	16.50	1030	52.16	107.02	201	0.49
**P112**	56.74	15.11	28.15	43.26	1926	7.25	1021	72.61	122.52	203	0.59
**P113**	74.13	3.51	22.36	25.87	2795	5.56	1025	41.34	60.89	205	0.68
**P125**	77.50	8.34	14.16	22.50	2000	nd	-	23.45	74.15	240	0.32
**P141**	69.12	7.38	23.50	30.88	2098	13.24	1012	46.73	104.77	225	0.45
**P146**	57.60	12.90	29.50	42.40	2000	4.72	1038	22.18	69.53	224	0.32
**P151**	64.42	10.08	25.50	35.58	2018	3.94	1015	52.27	112.67	225	0.46

**P155**	59.75	13.05	27.20	40.25	2024	22.70	1013	38.07	90.63	207	0.42
**P221**	82.82	4.98	12.20	17.18	2410	3.48	1006	109.74	122.94	253	0.89
**P228**	50.51	16.57	32.92	49.49	2644	5.94	1027	99.10	123.16	204	0.80
**P229**	74.06	2.47	23.47	25.94	2430	6.88	1032	29.45	86.99	222	0.34
**P233**	85.50	2.24	12.26	14.50	2682	3.86	1062	69.68	134.52	230	0.52
**P246**	74.34	4.62	21.04	25.66	1407	nd	-	58.96	89.18	205	0.66
**mean P**	**68.56***	**9.86**	**21.59***	**31.44***	**2040.26**	**9.55**	**1063.56**	**71.32**	**109.03**	**222.58**	**0.64**
SD	***13.59***	***6.68***	***8.82***	***13.59***	***398.41***	***6.29***	***122.28***	***30.82***	***22.74***	***19.02***	***0.20***

Table S3. Results of 5mC and 5hmC measurements in three subpopulations of spermatozoa fractionated with respect to their chromatin protamination status. Mean P-values statistically significant ($p<0.05$) from mean C values are marked with * for $p<0.05$, and ** for $p<0.01$. n = total number of spermatozoa analyzed in each case (at least 50 per population).

	**5mC [IF i.u.]**					**5hmC [IF i.u.]**					**5mC/5hmC ratio**			
**Control group n=10**	**Protaminated [pink]**	**semi-protaminated [purple]**	**Deprotaminated [navy blue]**	**mean of semi- and deprotaminated**	**n**	**Protaminated [pink]**	**semi-protaminated [purple]**	**Deprotaminated [navy blue]**	**mean of semi- and deprotamnated**	**n**	**Protaminated subpopulation**	**semi-protaminated sub-population**	**Deprotaminated subpopulation**	**mean of semi- and deprotaminated**
**C50**	135.46	75.13	51.59	63.36	223	145.77	122.78	108.36	115.57	219	0.93	0.61	0.48	0.55
**C52**	90.01	48.82	32.65	40.74	216	136.25	103.76	98.23	101.00	184	0.66	0.47	0.33	0.40
**C53**	97.23	45.62	28.91	37.27	244	108.28	94.87	90.06	92.47	236	0.90	0.48	0.32	0.40
**C55**	150.67	62.75	52.25	57.50	210	164.77	127.76	112.33	120.05	215	0.91	0.49	0.47	0.48
**C56**	128.59	65.04	47.24	56.14	211	120.22	105.15	93.01	99.08	228	1.07	0.62	0.51	0.57
**C57**	137.15	53.96	33.32	43.64	211	129.56	100.87	88.20	94.54	235	1.06	0.53	0.38	0.46
**C58**	133.52	92.96	31.88	62.42	209	142.67	116.99	107.99	112.49	213	0.94	0.79	0.30	0.55
**C59**	108.71	46.30	33.98	40.14	212	133.50	102.20	89.16	95.68	216	0.81	0.45	0.38	0.42
**C60**	106.84	46.59	32.17	39.38	226	131.55	108.96	89.44	99.20	217	0.81	0.43	0.36	0.40
**C61**	114.64	52.03	35.04	43.54	215	130.04	125.13	110.33	117.73	211	0.88	0.42	0.32	0.37
**mean C**	**120.28**	**58.92**	**37.90**	**48.41**	**220.80**	**134.26**	**110.85**	**98.71**	**104.78**	**216.40**	**0.90**	**0.53**	**0.38**	**0.46**
SD	***19.65***	***15.41***	***8.84***	***10.23***	***13.95***	***15.12***	***11.48***	***9.97***	***10.51***	***19.86***	***0.12***	***0.12***	**0.07**	**0.07**
**Patients' group (P) n=31**														
**P2**	113.10	60.90	68.36	64.63	182	136.49	134.54	120.79	127.67	163	0.77	0.45	0.57	0.51
**P3**	127.17	80.34	53.22	66.78	354	157.83	129.60	99.55	114.58	317	0.81	0.62	0.53	0.58
**P12**	107.19	82.28	64.82	73.55	172	152.76	142.54	124.70	133.62	165	0.68	0.58	0.52	0.55
**P14**	118.78	84.35	53.74	69.05	202	153.46	130.57	98.21	114.39	182	0.77	0.65	0.55	0.60
**P18**	111.15	76.60	48.39	62.50	235	134.57	106.79	75.73	91.26	237	0.83	0.72	0.64	0.68
**P19**	97.33	57.97	34.56	46.27	158	134.70	109.49	75.91	92.70	173	0.72	0.53	0.46	0.50
**P22**	109.88	77.91	59.83	68.87	183	125.35	102.44	82.64	92.54	201	0.88	0.76	0.72	0.74
**P24**	63.55	55.03	36.47	45.75	168	134.52	121.77	103.81	112.79	157	0.47	0.45	0.35	0.41
**P52**	151.56	115.36	85.54	100.45	165	153.67	128.90	105.27	117.09	175	0.99	0.89	0.81	0.86
**P65**	59.60	34.16	16.78	25.47	168	123.33	97.57	69.46	83.52	155	0.48	0.35	0.24	0.30
**P67**	104.17	66.92	37.85	52.39	206	135.67	108.25	81.73	94.99	207	0.77	0.62	0.46	0.55
**P72**	46.64	22.15	22.54	22.35	178	85.03	60.10	52.91	56.51	175	0.55	0.37	0.43	0.40

**P73**	117.04	85.96	64.30	75.13	183	90.83	78.53	62.00	70.27	174	1.29	1.09	1.04	1.07
**P75**	108.48	55.36	32.76	44.06	199	133.28	111.48	70.78	91.13	183	0.78	0.50	0.46	0.48
**P77**	156.50	125.35	99.75	112.55	173	139.64	122.89	111.94	117.42	187	1.12	1.02	0.89	0.96
**P88**	96.00	60.23	44.45	52.34	192	125.39	87.04	74.75	80.90	194	0.77	0.69	0.59	0.65
**P90**	70.78	41.59	22.14	31.87	168	110.09	91.96	74.02	82.99	193	0.64	0.45	0.30	0.38
**P110**	66.95	48.55	30.97	39.76	171	100.39	81.36	67.21	74.29	160	0.67	0.60	0.46	0.54
**P111**	42.68	50.01	49.49	49.75	169	109.46	106.46	93.00	99.73	182	0.39	0.47	0.53	0.50
**P112**	94.94	56.78	48.90	52.84	187	130.55	117.34	102.81	110.08	183	0.73	0.48	0.48	0.48
**P113**	49.53	33.21	28.64	30.93	175	76.30	50.33	36.43	43.38	153	0.65	0.66	0.79	0.71
**P125**	27.83	18.80	19.94	19.37	174	84.38	60.35	55.50	57.93	185	0.33	0.31	0.36	0.33
**P141**	54.24	50.13	31.51	40.82	174	113.23	99.11	86.78	92.95	162	0.48	0.51	0.36	0.44
**P146**	23.71	22.03	19.94	20.99	213	71.66	67.44	55.70	61.57	229	0.33	0.33	0.36	0.34
**P151**	58.05	47.04	41.55	44.30	225	119.53	109.28	100.08	104.68	223	0.49	0.43	0.42	0.42
**P155**	46.97	30.94	27.59	29.27	178	100.85	66.51	81.13	73.82	189	0.47	0.47	0.34	0.40
**P221**	124.52	81.98	52.00	66.99	321	133.81	104.68	74.85	89.77	339	0.93	0.78	0.69	0.75
**P228**	130.26	91.52	53.29	72.41	170	128.37	107.51	79.31	93.41	172	1.01	0.85	0.67	0.78
**P229**	44.93	25.43	15.38	20.41	156	111.59	79.34	42.27	60.81	184	0.40	0.32	0.36	0.34
**P233**	98.80	63.47	35.55	49.51	379	145.78	135.28	109.35	122.32	382	0.63	0.47	0.33	0.40
**P246**	83.74	43.24	24.48	33.86	182	104.24	74.26	58.95	66.61	201	0.80	0.58	0.42	0.51
**mean P**	**87.29***	**59.54**	**42.73**	**51.13**	**198.71**	**121.20**	**100.76**	**81.53**	**91.15**	**199.42**	**0.70****	**0.58**	**0.52***	**0.55**
SD	***36.01***	***26.34***	***20.11***	***22.72***	***53.59***	***23.42***	***24.95***	***22.39***	***23.03***	***52.72***	***0.23***	***0.20***	**0.19**	**0.19**

Table S4 Analysis of correlations between all analyzed parameters in Control and Patients' groups. Statistical description: *** p<0.0001, ** 0.0001<p<0.01,* <0.01p<0.05, ns - statistically non-significant (p>0.05), #ns 0.05<p<0.06 (statistically non-significant but at the border value). Correlation coefficients: R^2 for linear regression and Pearson *r* values included for statistically significant and border results.

Control Group (C) / Patients' Group (P)		sperm chromatin integrity [%]		unfractionated total sperm population			properly protaminated subpopulation			semen parameters					
														motility [%]	
		protaminated	with fragmented DNA	global 5mC	global 5hmC	5mC/5hmC ratio	mean 5mC	mean 5hmC	5mC/5hmC ratio	concentration [x106/mL]	volume [mL]	total count [x106]	morphology [%]	progressive	total
sperm chromatin integrity [%]	protaminated		ns	**<br>R^2 0.4294<br>*r* -0.6946	*<br>R^2 0.0936<br>*r* -0.3975	*<br>R^2 0.1615<br>*r* -0.4346	**<br>R^2 0.0634<br>*r* -0.7746	**<br>R^2 0.7383<br>*r* -0.6848	ns	ns	ns	*<br>R^2 0.1342<br>*r* -0.4696	ns	ns	ns
	with fragmented DNA	ns		ns	ns	ns	ns	ns	ns	ns	ns	ns	ns	ns	ns
unfractionated total sperm population	global 5mC	ns	ns		**<br>R^2 0.3192<br>*r* 0.6667	***<br>R^2 0.4392<br>*r* 0.5236	ns	ns	ns	ns	ns	#ns<br>R^2 0.0259<br>*r* 0.3990	ns	ns	ns
	global 5hmC	ns	ns	***<br>R^2 0.5853<br>*r* 0.7340		ns	**<br>R^2 0.6358<br>*r* 0.8546	ns	*<br>R^2 0.5495<br>*r* 0.9427	ns	ns	ns	ns	ns	ns
	5mC/5hmC ratio	ns	ns	***<br>R^2 0.8269<br>*r* 0.9093	**<br>R^2 0.2076<br>*r* 0.4557		ns	ns	ns	ns	ns	ns	ns	ns	ns
properly protaminated subpopulation	mean 5mC	ns	ns	***<br>R^2 0.9308<br>*r* 0.9117	***<br>R^2 0.5422<br>*r* 0.7684	***<br>R^2 0.8025<br>*r* 0.8958		#ns<br>R^2 0.3690<br>*r* 0.3300	*<br>R^2 0.5028<br>*r* 0.9412	ns	ns	ns	ns	ns	#ns<br>R^2 0.1368<br>*r* 0.8609
	mean 5hmC	ns	ns	***<br>R^2 0.5892<br>*r* 0.7407	***<br>R^2 0.8960<br>*r* 0.9033	**<br>R^2 0.2332<br>*r* 0.4829	***<br>R^2 0.5661<br>*r* 0.7556		ns	#ns<br>R^2 0.0121<br>*r* 0.8587	ns	ns	ns	ns	ns
	5mC/5hmC ratio	ns	ns	***<br>R^2 0.6764<br>*r* 0.7681	**<br>R^2 0.1812<br>*r* 0.4709	***<br>R^2 0.8569<br>*r* 0.9299	***<br>R^2 0.8019<br>*r* 0.8827	*<br>R^2 0.1688<br>*r* 0.3882		ns	ns	ns	ns	ns	#ns<br>R^2 0.2653<br>*r* 0.8601
semen parameters	concentration [x10[6]/mL]	ns	ns	ns	ns	ns	ns	ns	ns		*<br>R^2 0.2028<br>*r* -0.4503	***<br>R^2 0.4493<br>*r* 0.6703	ns	*<br>R^2 0.3462<br>*r* 0.5884	*<br>R^2 0.2008<br>*r* 0.4481
	volume [mL]	ns	ns	ns	ns	ns	ns	ns	ns	ns		ns	ns	ns	ns
	total count [x10[6]]	ns	ns	ns	ns	ns	ns	ns	ns	***<br>R^2 0.4918<br>*r* 0.7013	**<br>R^2 0.2194<br>*r* 0.4684		ns	ns	ns
	morphology [%]	ns	ns	*<br>R^2 0.1690<br>*r* 0.4111	ns	**<br>R^2 0.2097<br>*r* 0.4579	**<br>R^2 0.2102<br>*r* 0.4585	ns	**<br>R^2 0.2579<br>*r* 0.4883	ns	ns	#ns<br>R^2 0.1208<br>*r* 0.3475		*<br>R^2 0.2955<br>*r* 0.5436	**<br>R^2 0.2976<br>*r* 0.5455

	**motility [%]**	**progressive**	ns	ns	ns	ns	ns	ns	ns	ns	ns	ns	ns	ns		**<br>R^2 0.7565<br>*r* 0.8697
		**total**	ns	ns	ns	ns	ns	ns	***<br>R^2 0.0427<br>*r* -0.2067	ns	ns	ns	ns	ns	***<br>R^2 0.9349<br>*r* 0.9669	

Table S5 Literature data concerning methylation analysis of sperm DNA in fertile and infertile males. N – normozoospermia, O – oligozoospermia, A – asthenozoospermia, T – teratozoospermia, IVF – in vitro fertilization, ICSI – intracytoplasmic sperm injection, P1, P2 – protamine 1, 2, IF – immunofluorescence, IHC – immunohistochemistry, ELISA - enzyme-linked immunosorbent assay, HPLC – high pressure liquid chromatography, TLC – thin-layer chromatography, UPLC-MS/MS – ultra performance liquid chromatography - tandem mass spectrometry

observations	method of methylation evaluation	seminology/ infertility	ref.
methylation changes in oligozoospermia; correlations with sperm count, motility, morphology	450 K epic chip; bisulfite PCR; genes	infertile: 101 O; controls: 64 N	[72]
hypermethylation of selected genes vs. age, oligozoospermia, numerical chromosome abnormalities, lower pregnancy rate	450K epic chip	infertile: 42 - 9 N, 9 O, 9 A, 12 T, 3 AT; controls: 19 N	[59]
decreased methylation in spermatozoa used in IVF and resulted in poor blastocysts	450K epic chip	infertile: 40 N IVF	[34]
sperm DNA methylation stable between semen samples collection	450K epic chip	controls: 12 N	[123]
specific CpGs methylation linked to astheno- and teratozoospermia	450K epic chip	infertile: 15 O, 27 T, 20 A; controls: 79 N	[66]
genomic loci with altered DNA correlated to decreased fecundity	450K epic chip	infertile: 29 N; controls: 27 N	[67]
poor-quality spermatozoa (Percoll gradient) have altered methylation	450K epic chip	infertile: 20 N	[65]
specific CpGs methylation changes in infertile patients	450K epic chip	infertile: 15 OA; controls: 15 N	[71]
correlation between methylation changes and reduced sperm count and motility	450K epic chip; bisulfite PCR MiSeq sequencing	infertile: O/OA 6-32; control: N 6-20	[77]
sperm motility correlated to methylation pattern	bisulfite PCR; gene	infertile: 25 A; control: 27N	[113]
hypermethylation of MTHFR associated with idiopathic infertility; higher methylation in oligozoospermia	bisulfite PCR; gene	infertile: O 64, N 30; control: N 54	[75]
correlations: hypermethylation vs. low motility, abnormal morphology	bisulfite PCR; genes	infertile: 27; control: 11 N with female side infertility	[69]
changed methylation in oligozoospermia	bisulfite PCR; genes	infertile: 10 O; controls: 29 N	[120]
altered methylation in some patients	bisulfite PCR; genes	infertile: 20 P1/P2; controls: 10 N	[38]
correlation between methylation and ROS level	bisulfite PCR; genes; imprinting	infertile: 151 N	[99]
increased methylation in 6/7 imprinted genes in oligo- and protamines' patients	bisulfite PCR; genes; imprinting	infertile: 8-13 O, 9-11 P1:P2; controls: 5-7 N	[37]
imprinting changed in oligozoospermia; global 5mC normal	bisulfite PCR; genes; imprinting	97 infertile: 79 N, 18 O	[107]
imprinting errors in oligozoospermia	bisulfite PCR; genes; imprinting	infertile: 151 N, 70 O	[108]
trend of methylation changes in severe oligozoospermia	bisulfite PCR; genes; imprinting	infertile: 44 O; controls: 9 N	[112]
imprinting errors in oligozoospermia	bisulfite PCR; genes; imprinting	infertile: 14 OAT, 20 OT; controls: 5 N	[97]
methylation changes in oligozoospermia	bisulfite PCR; genes; imprinting	infertile: 175 O, 119 N	[74]
lower methylation in imprinted genes in infertile males, especially oligozoospermic	bisulfite PCR; genes; imprinting	idiopathic: 39 N, 96 O; control: 59 N	[116]
methylation in specific CpGs different between low motile astheno- sperm vs. high motile normozoospermic cells	bisulfite sequencing; genes	infertile: 7 A; controls: 8 N	[111]

the overestimated prevalence of aberrant methylation in swim-up purified sperm of infertile men;	deep bisulfite sequencing and whole methylome analysis	infertile: 93 OAT	[124]
sperm 5mC increasing with age; 5hmC lower in sperm than in blood	ELISA	controls: 37 N	[64]
correlation between methylation vs. sperm concentration, motility, low genome integrity	ELISA	infertile: 30 OA, 62 N	[70]
methylation and histone retention decreased in selected spermatozoa; better motility in selected fraction	ELISA; 3x 720 K MeDIP-chip	infertile: 52 A/OA; control: 41 N	[109]
disrupted methylation in patients	HM27 BeadChip assay; bisulfite pyrosequencing	infertile: 15 P1/P2, 13 IVF/ICSI; control: 15 N	[25]
low motility samples with changed methylation profile	HM27 BeadChip assay; imprinting	donors: 21	[118]
apoptotic sperm cells' production linked to disordered spermatogenesis, leading to DNA hypermethylation	HPLC; cytometry; IHC	controls: 16 N	[80]
no link between protamines' level and 5mC	IF	195 P1/P2	[91]
correlation between methylation and abnormal morphology (hypomethylation)	IF	control: 10 N with female side infertility	[110]
decreased methylation in swim up fraction; positive correlation between 5mC level in swim up fraction vs. DFI	IF	controls: 50 N	[132]
negative correlation 5mC vs. SCD test; no correlation fertlilization rate vs. 5mC	IF	66 ICSI and 35 IVF pairs	[101]
negative correlation 5mC vs. TUNEL, ROS	IF	infertile N 45	[102]
low chromatin integrity and high hypomethylation in carriers of chromosome structural aberrations vs. hypermethylation in controls	IF; TLC	infertile: 24 chromosome aberration carriers: 13 with decreased seminology,11 N; controls: 23 N	[42]
increased DNA damage when hypomethylated in varicocele patients	IF/cytometry	infertile with varicocele: 44; control: 15 N	[92]
no link between methylation and seminology	IF/cytometry	IVF patients: 63, incl. O, A (but no details)	[45]
methylation decreased in astheno- and teratozoospermia	IHC	IVF patients: 10 N, 13 incl. O, A, T, OA, OAT	[44]
sperm motility correlated to methylation pattern	massArray; genes	inferttile: 46 A; control: 49 N	[114]
increased methylation in decreased semen parameters; decreased in 90% percoll fraction	pyrosequencing	infertile: 60 P1/P2, 32 O; controls: 40 N	[121]
in OA abnormal methylation; in N homogenous methylation state	pyrosequencing; deep bisulfite sequencing; micromanipulation - 10 spermatozoa/case	infertile: 3 O, 5 T, 3 OA, 3 OT, 12 OAT; controls: 19 N	[122]
altered methylation pattern in genes in infertile men with low sperm quality	HM27 BeadChip assay; bisulfite pyrosequencing; Methylamp Global DNA methylation kit	infertile: 29 N; controls: 17 N	[88]
lower global DNA methylation level in sperm samples washed by PBS vs. density gradient centrifugation	UPLC-MS/MS	infertile: 24 (without any details)	[79]
